# Supplementary figures and images for: CLUH controls astrin-1 expression to couple mitochondrial metabolism to cell cycle progression
Source: eLife. 2022 May 13;11:e74552. doi: 10.7554/eLife.74552 (PMC9135405; doi:10.7554/eLife.74552)

Figure 1A

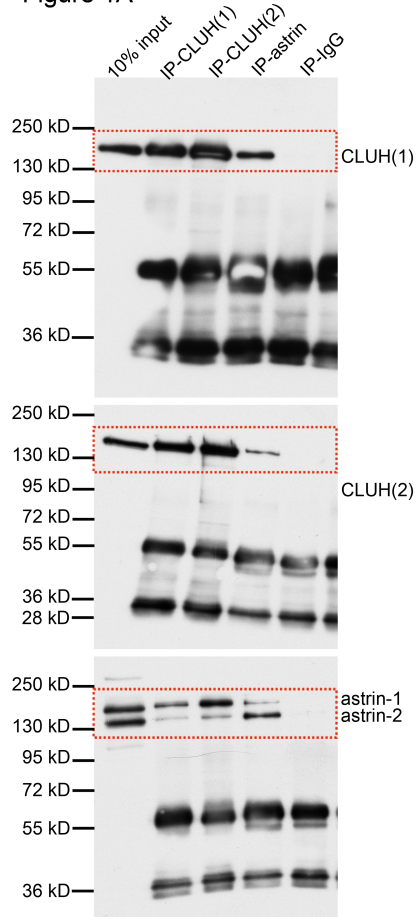

Figure 1B

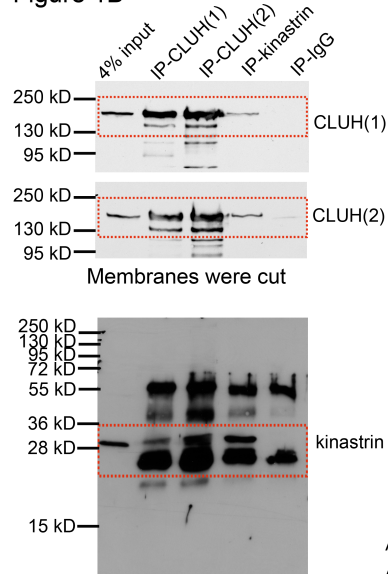

Figure 1D

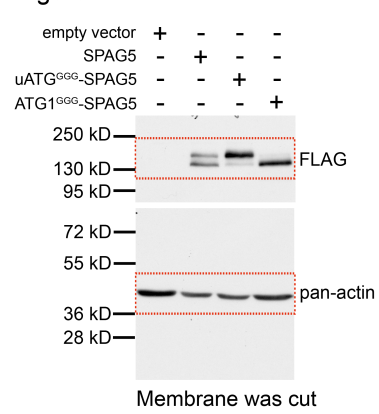

Figure 1E

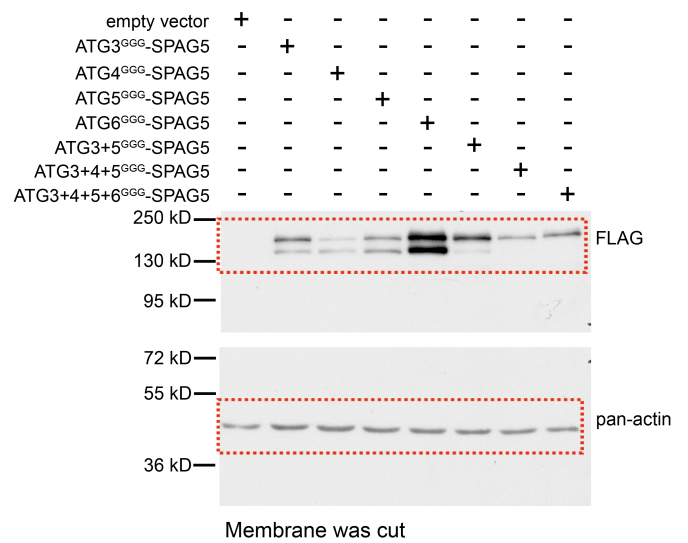

Figure 1F

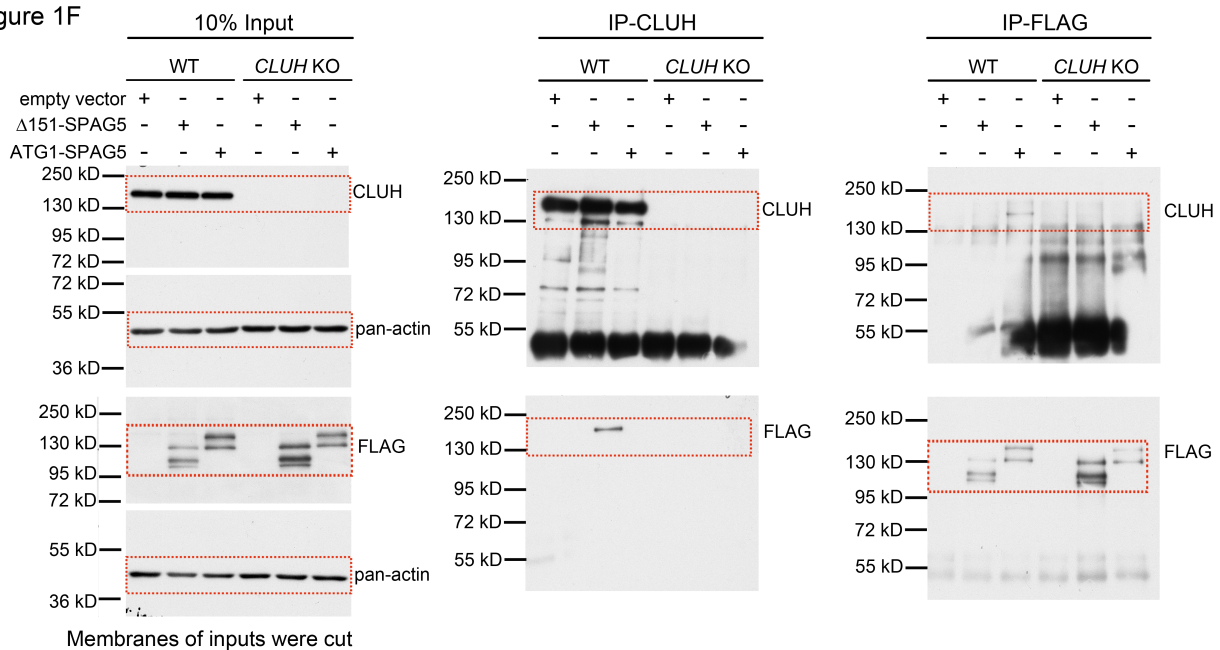

Supplement: Figure 1—source data 1. [file elife-74552-fig1-data1.pdf]

Figure 1H

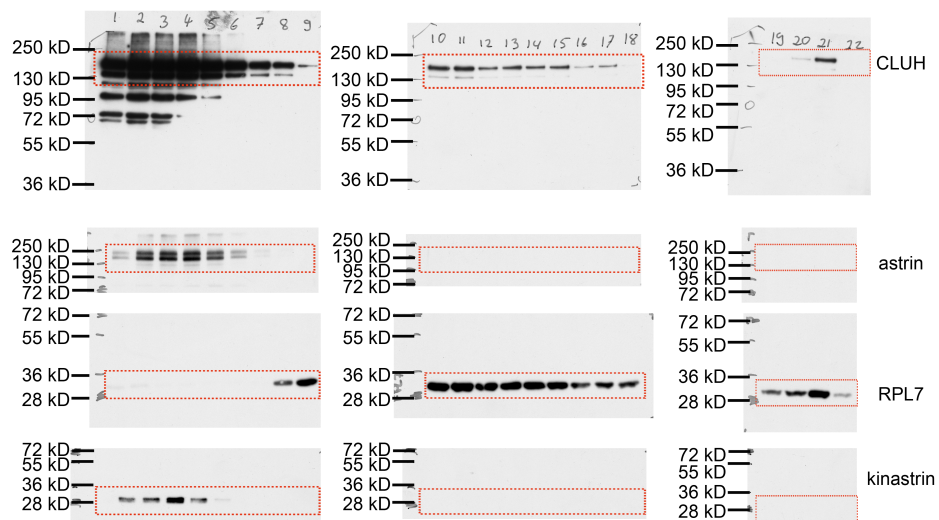

Supplement: Figure 1—source data 2. [file elife-74552-fig1-data2.pdf]

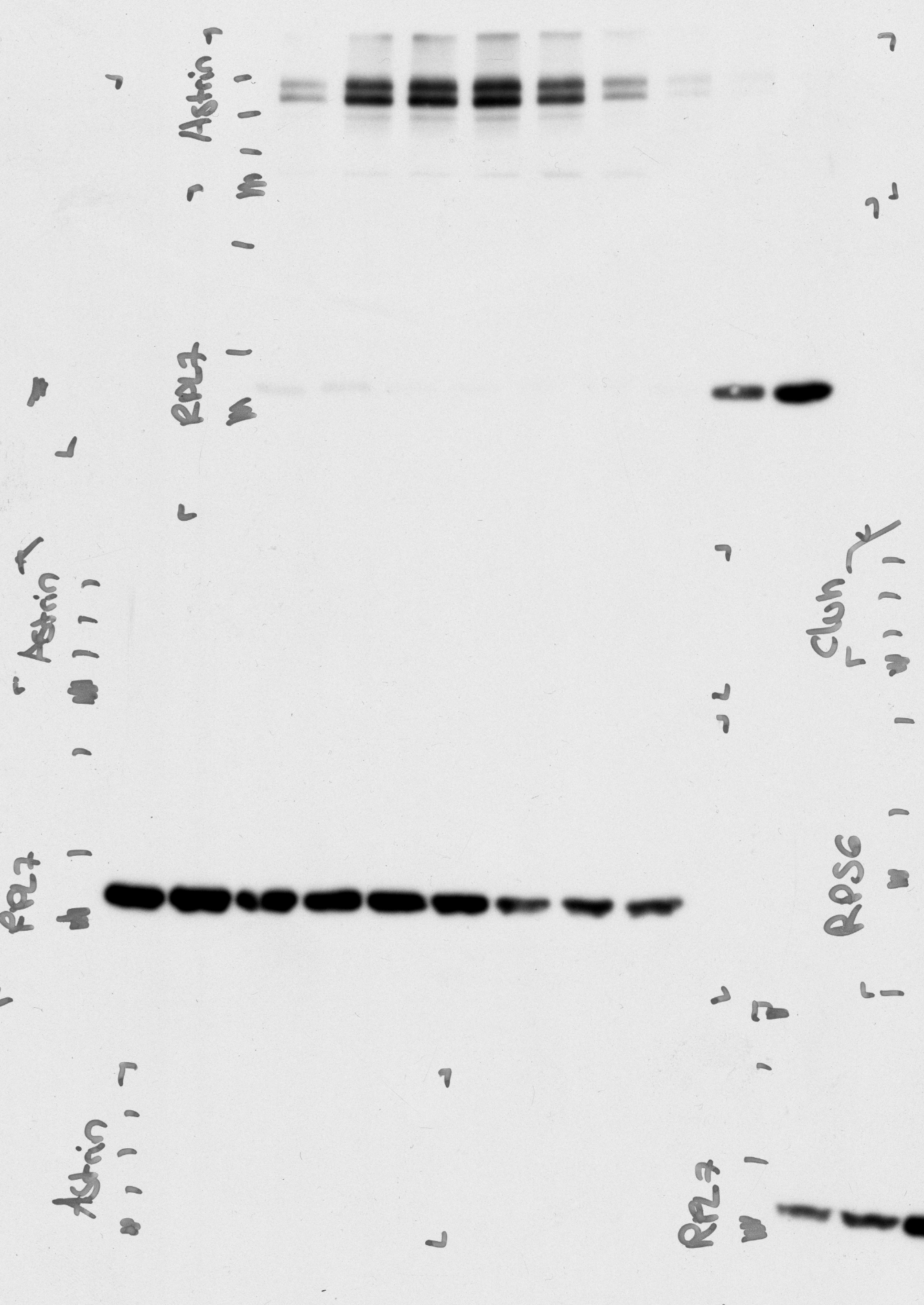

Supplement: Figure 1—source data 3. [file elife-74552-fig1-data3.zip › Figure 1ΓÇösource data 3/Uncropped blots for Figure 1H/Polysome Profiling astrin.tif]

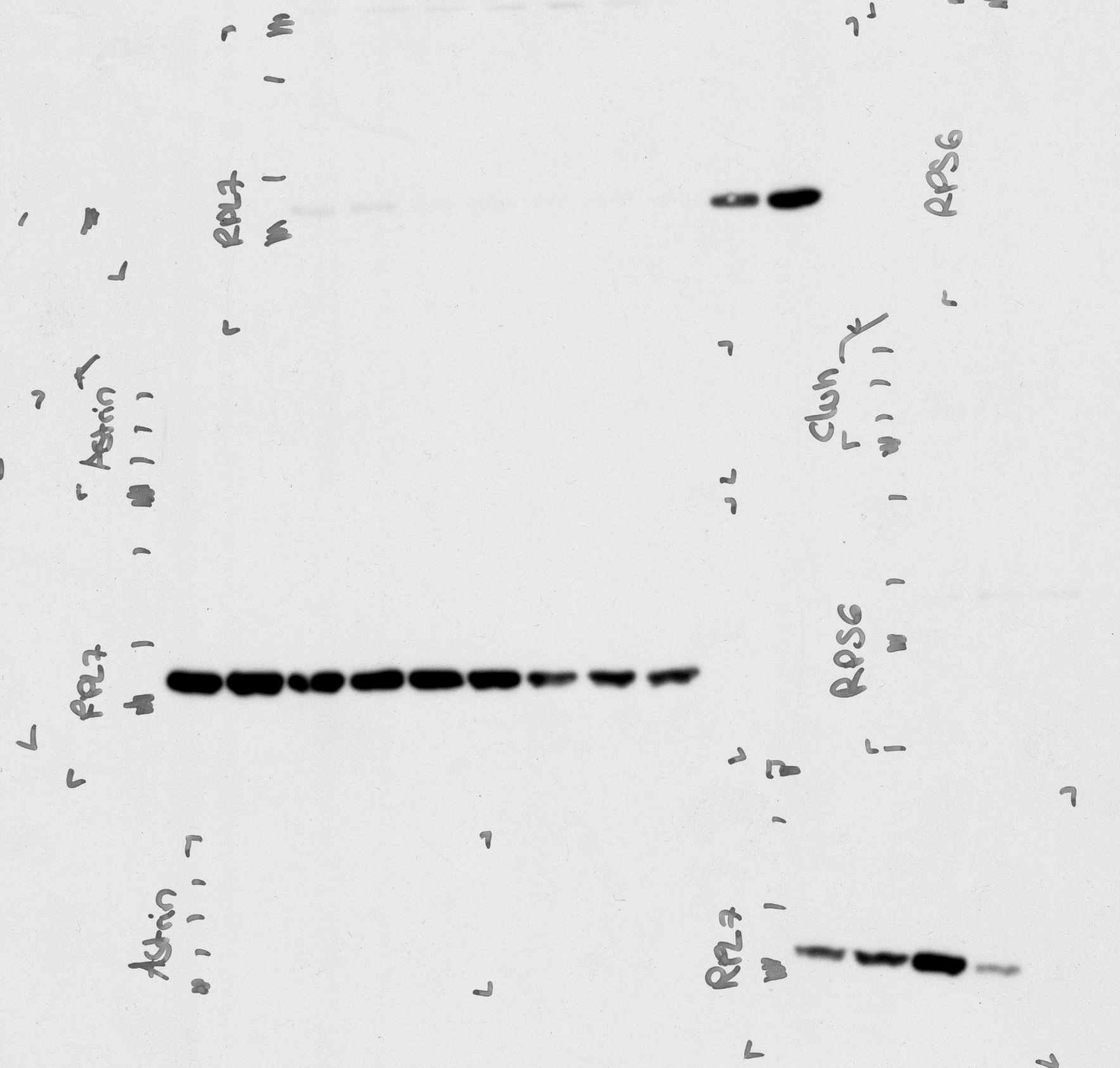

Supplement: Figure 1—source data 3. [file elife-74552-fig1-data3.zip › Figure 1ΓÇösource data 3/Uncropped blots for Figure 1H/Polysome Profiling RPL7.tif]

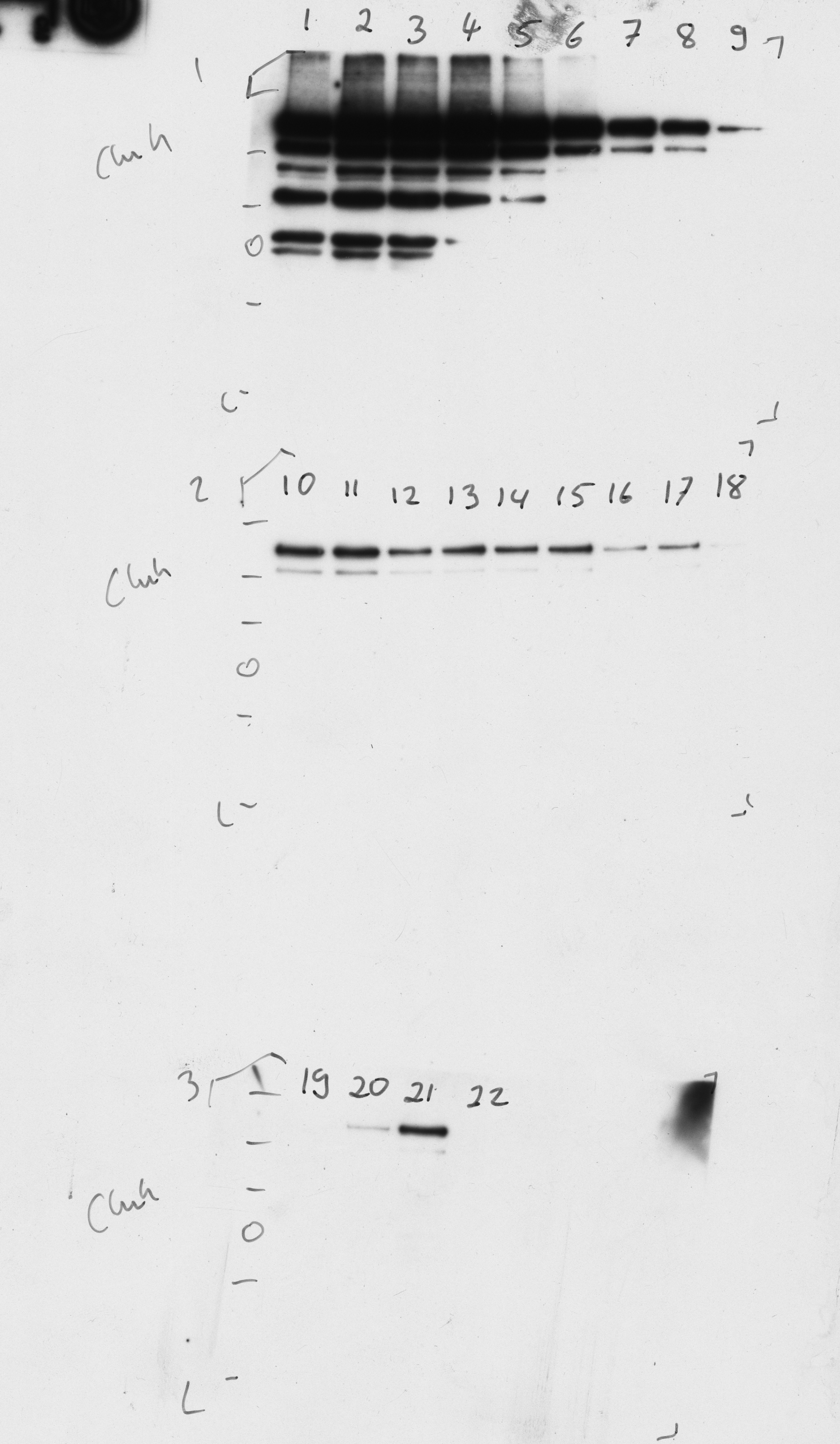

Supplement: Figure 1—source data 3. [file elife-74552-fig1-data3.zip › Figure 1ΓÇösource data 3/Uncropped blots for Figure 1H/Polysome Profiling CLUH.tif]

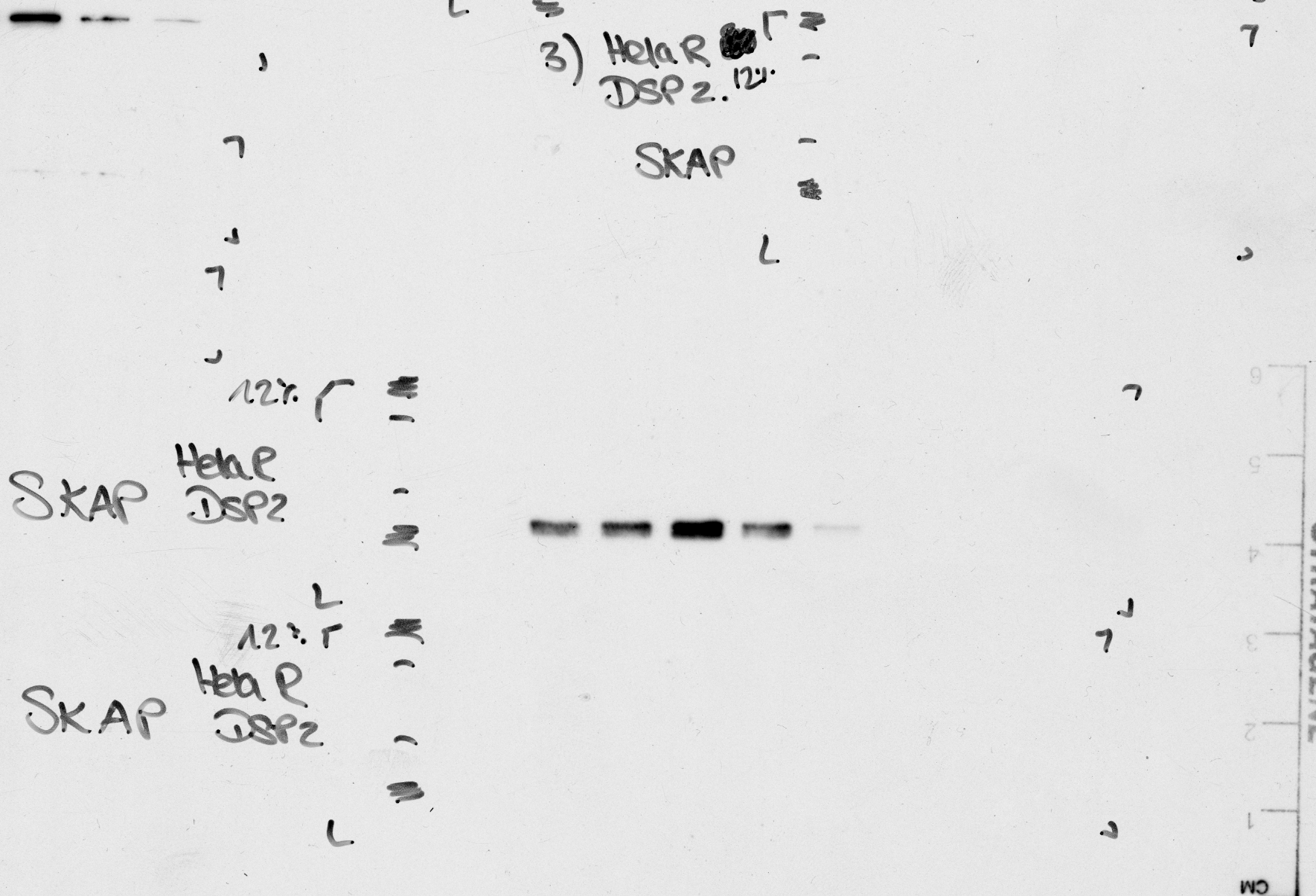

Supplement: Figure 1—source data 3. [file elife-74552-fig1-data3.zip › Figure 1ΓÇösource data 3/Uncropped blots for Figure 1H/Polysome Profiling kinastrin.tif]

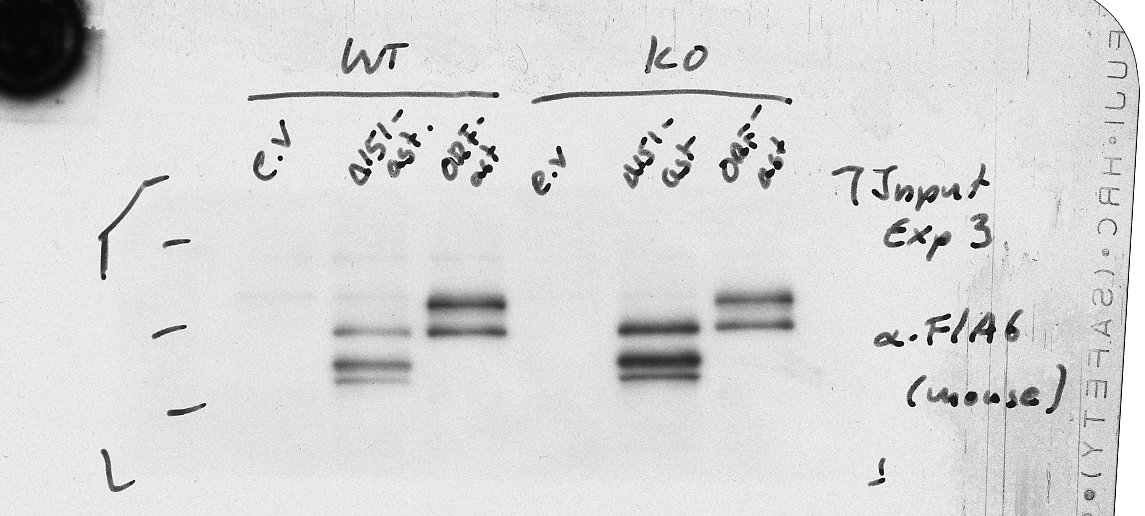

Supplement: Figure 1—source data 3. [file elife-74552-fig1-data3.zip › Figure 1ΓÇösource data 3/Uncropped blots for Figure 1F /Input FLAG.tif]

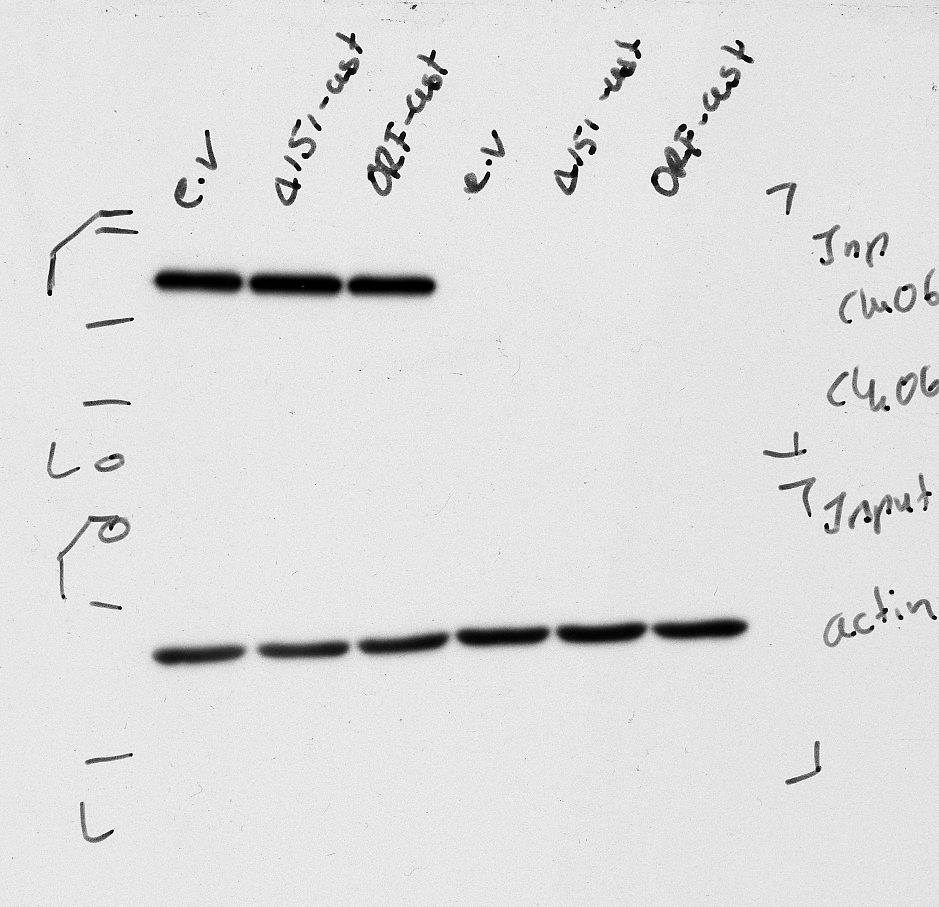

Supplement: Figure 1—source data 3. [file elife-74552-fig1-data3.zip › Figure 1ΓÇösource data 3/Uncropped blots for Figure 1F /Input CLUH and actin.tif]

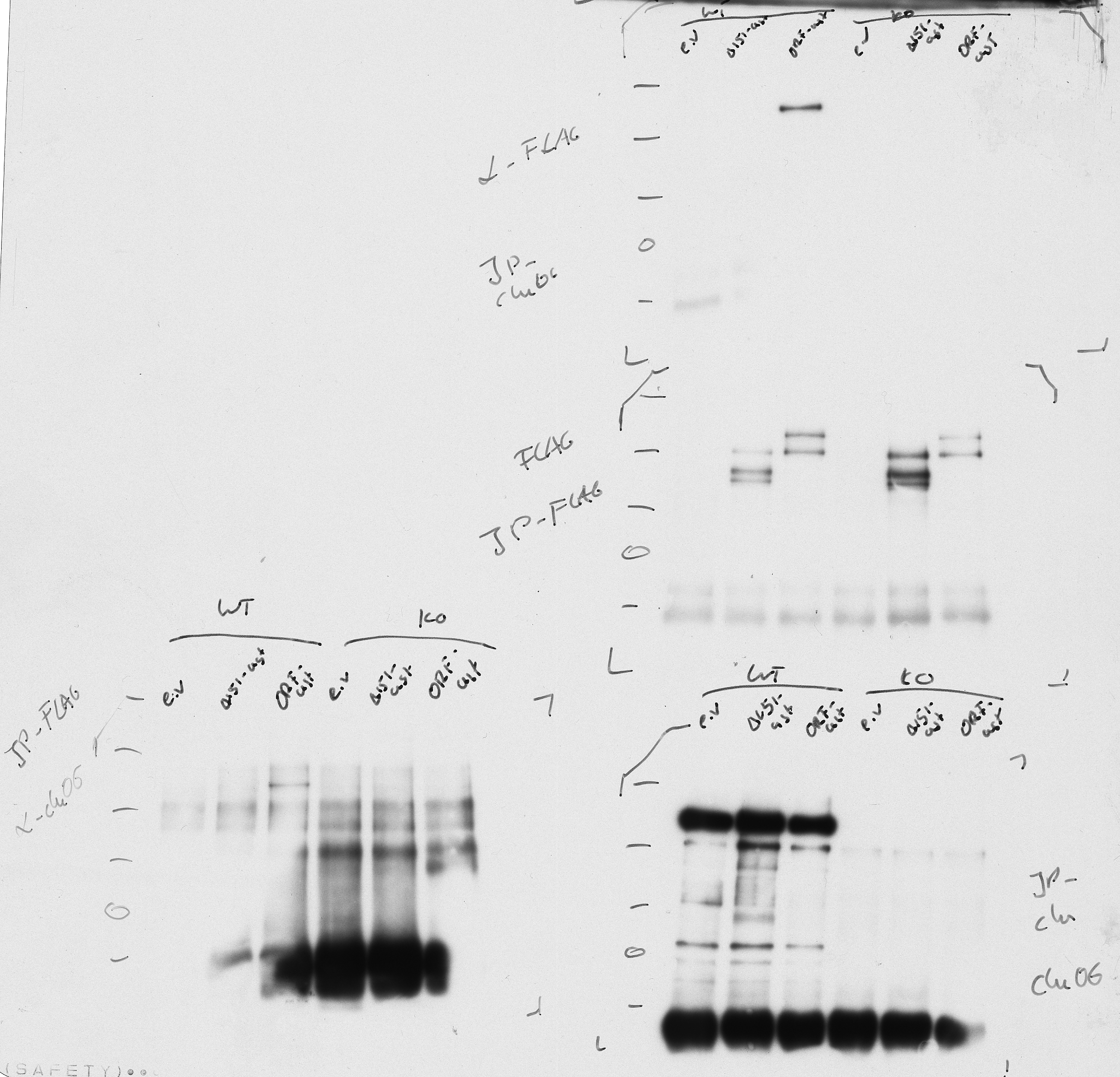

Supplement: Figure 1—source data 3. [file elife-74552-fig1-data3.zip › Figure 1ΓÇösource data 3/Uncropped blots for Figure 1F /IP FLAG and CLUH.tif]

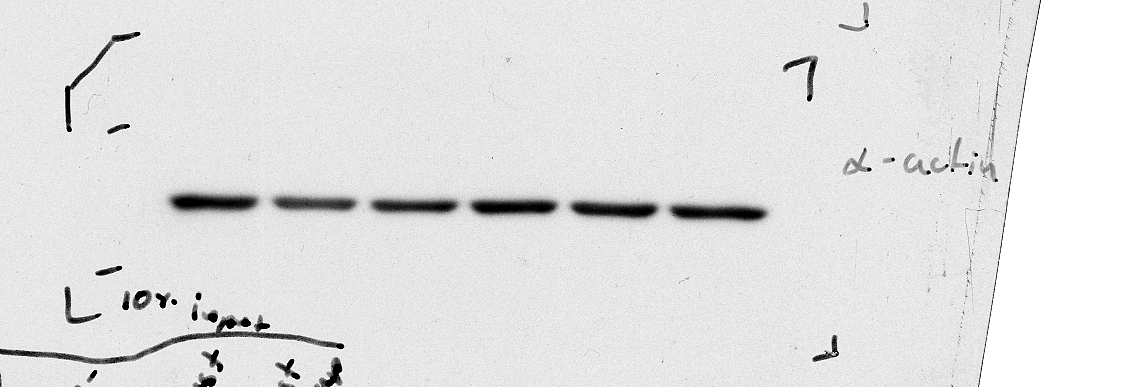

Supplement: Figure 1—source data 3. [file elife-74552-fig1-data3.zip › Figure 1ΓÇösource data 3/Uncropped blots for Figure 1F /Input FLAG actin.tif]

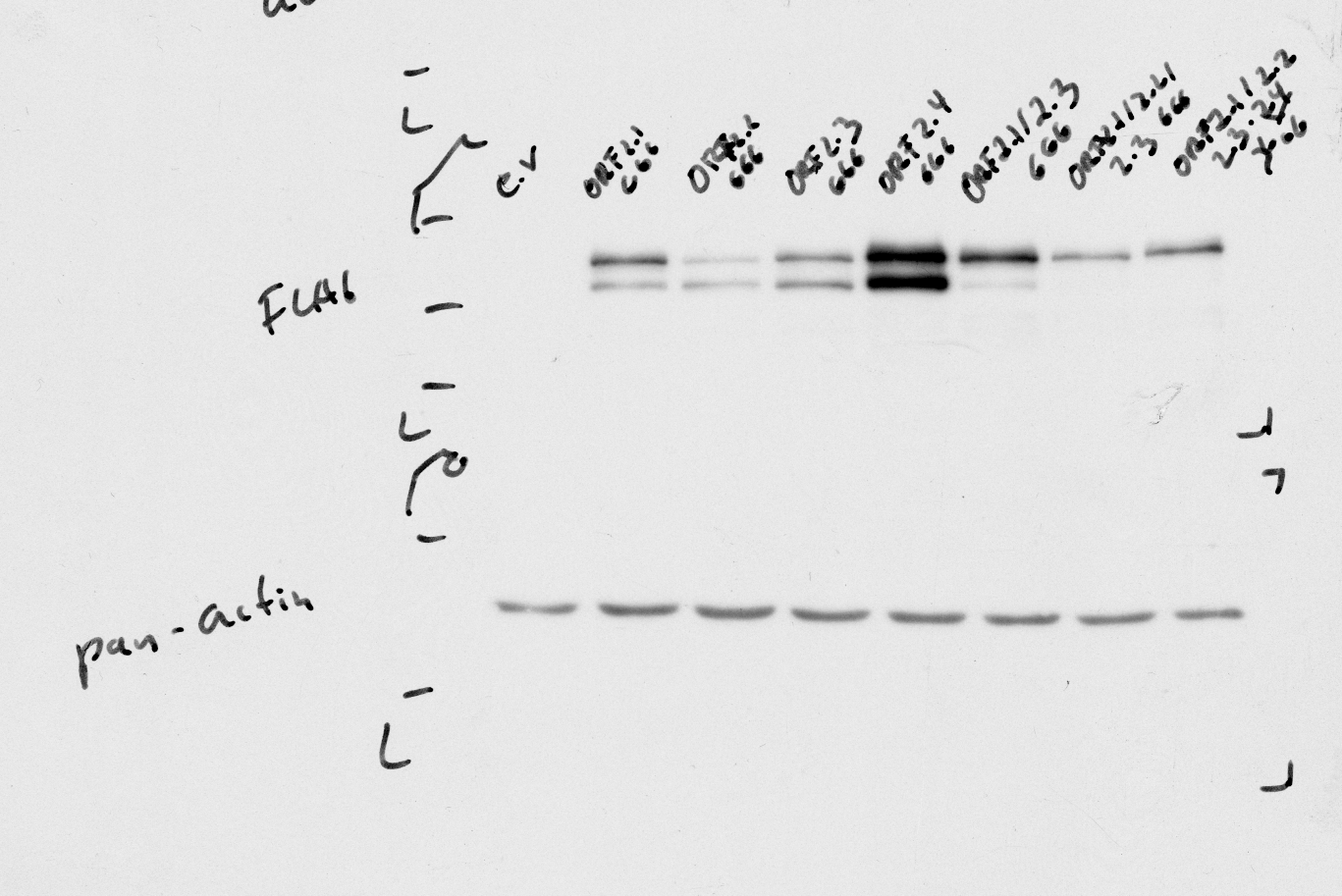

Supplement: Figure 1—source data 3. [file elife-74552-fig1-data3.zip › Figure 1ΓÇösource data 3/Uncropped blots for Figure 1E/OE astrin constructs FLAG and actin.tif]

Figure 1-figure supplement1

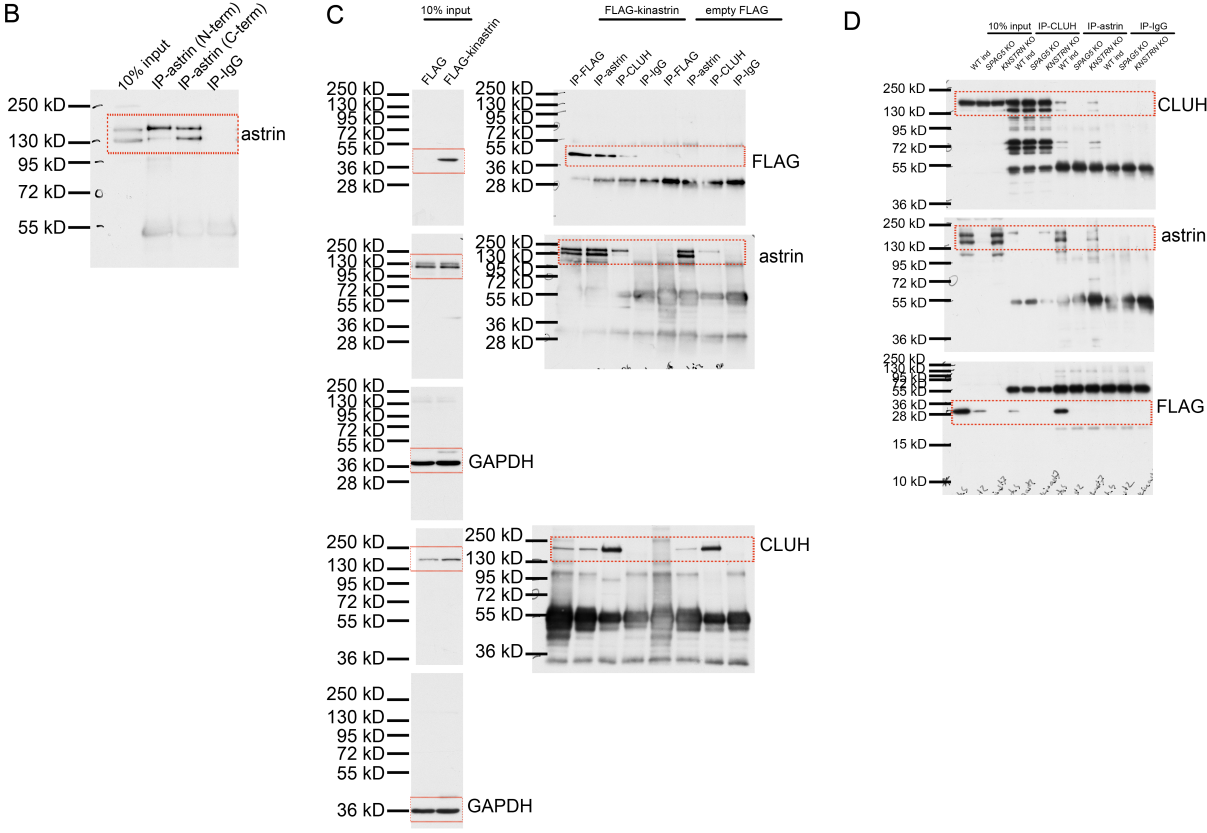

Supplement: Figure 1—figure supplement 1—source data 1. [file elife-74552-fig1-figsupp1-data1.pdf]

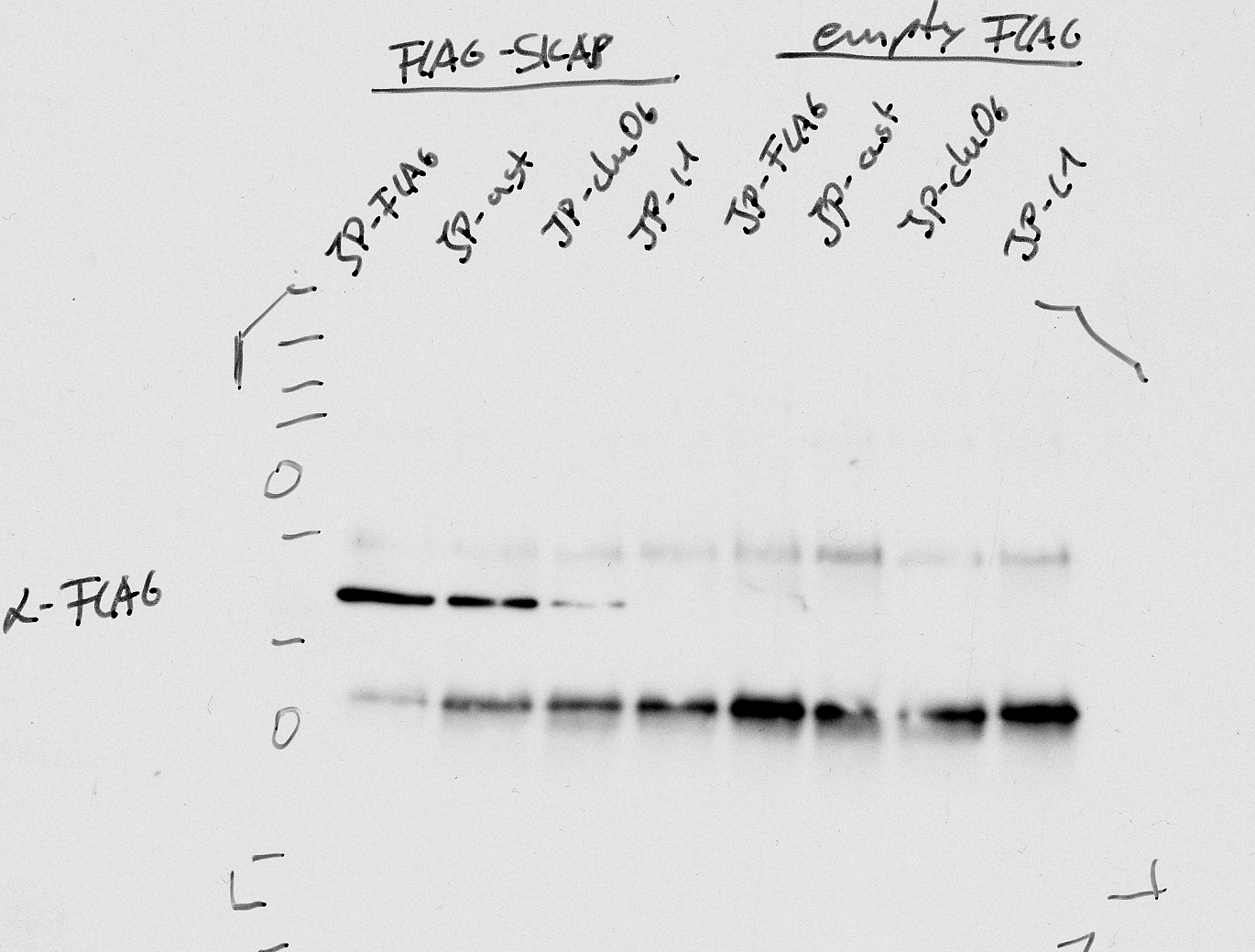

Supplement: Figure 1—figure supplement 1—source data 2. [file elife-74552-fig1-figsupp1-data2.zip › Figure 1-figure supplement 1-source data 2/Uncropped blots for Figure 1-Figure supplement 1C/HEK293T IP OE kinastrin FLAG.tif]

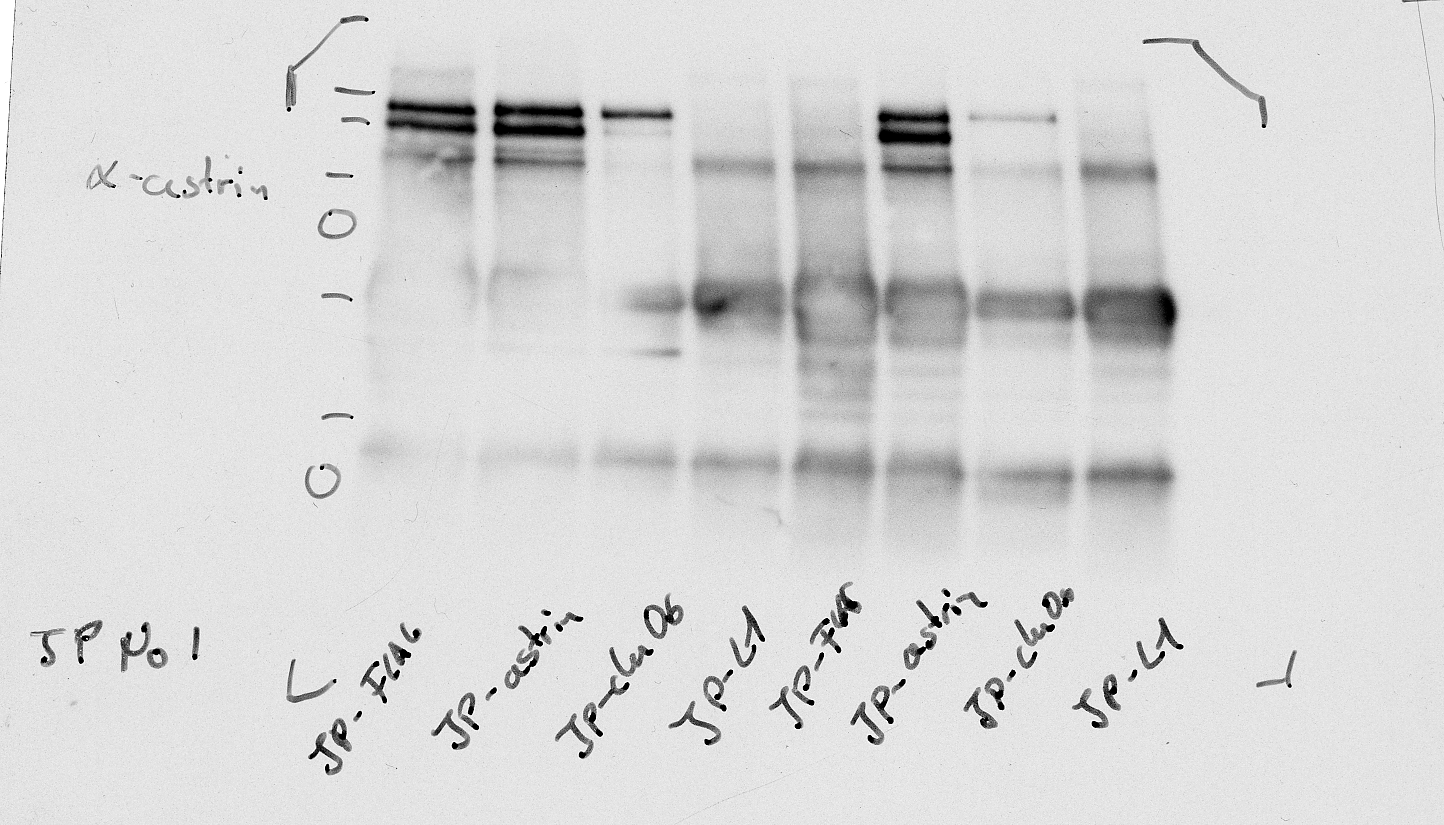

Supplement: Figure 1—figure supplement 1—source data 2. [file elife-74552-fig1-figsupp1-data2.zip › Figure 1-figure supplement 1-source data 2/Uncropped blots for Figure 1-Figure supplement 1C/HEK293T IP OE kinastrin astrin.tif]

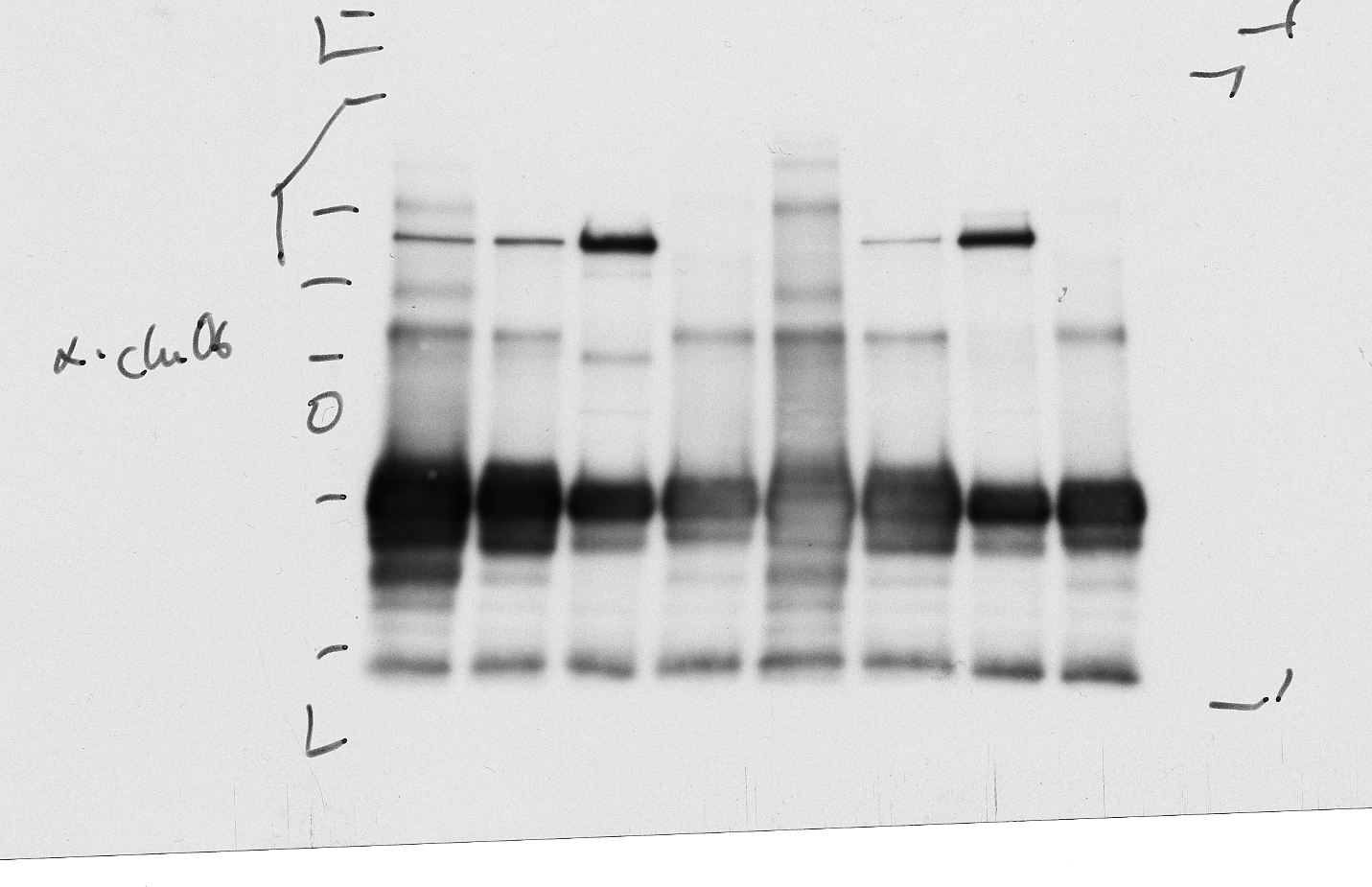

Supplement: Figure 1—figure supplement 1—source data 2. [file elife-74552-fig1-figsupp1-data2.zip › Figure 1-figure supplement 1-source data 2/Uncropped blots for Figure 1-Figure supplement 1C/HEK293T IP OE kinastrin CLUH.tif]

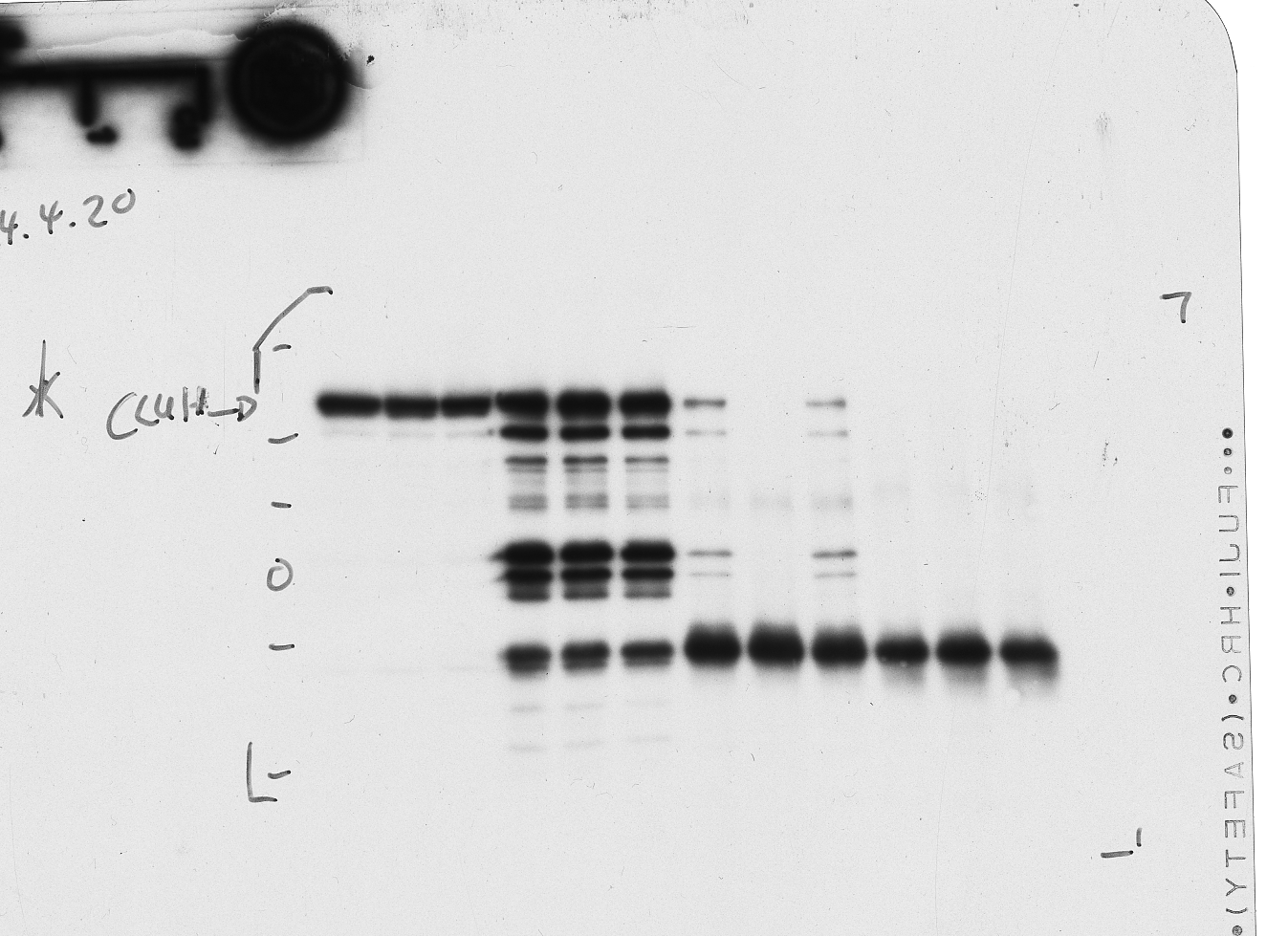

Supplement: Figure 1—figure supplement 1—source data 2. [file elife-74552-fig1-figsupp1-data2.zip › Figure 1-figure supplement 1-source data 2/Uncropped blots for Figure 1-Figure supplement 1D/IP CLUH.tif]

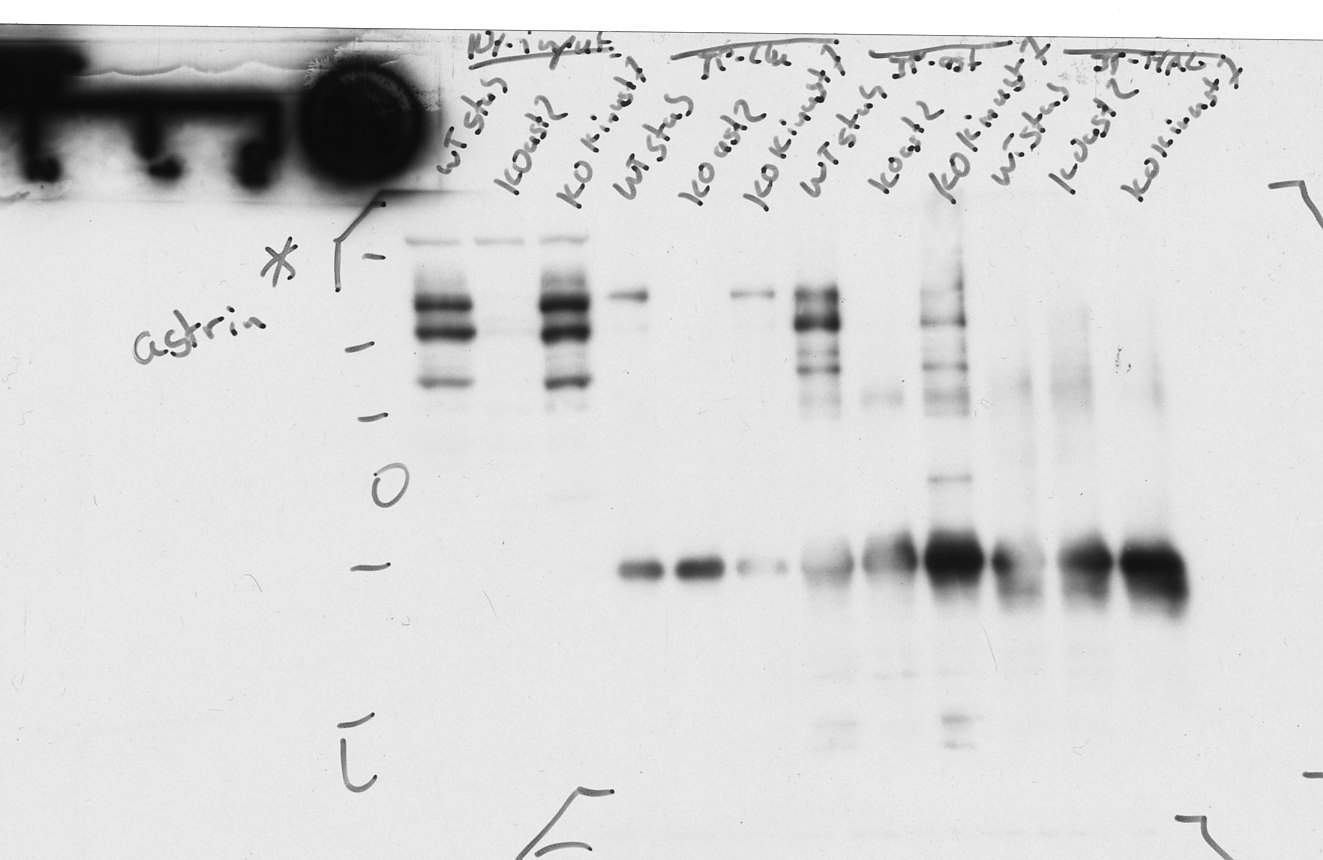

Supplement: Figure 1—figure supplement 1—source data 2. [file elife-74552-fig1-figsupp1-data2.zip › Figure 1-figure supplement 1-source data 2/Uncropped blots for Figure 1-Figure supplement 1D/IP astrin.tif]

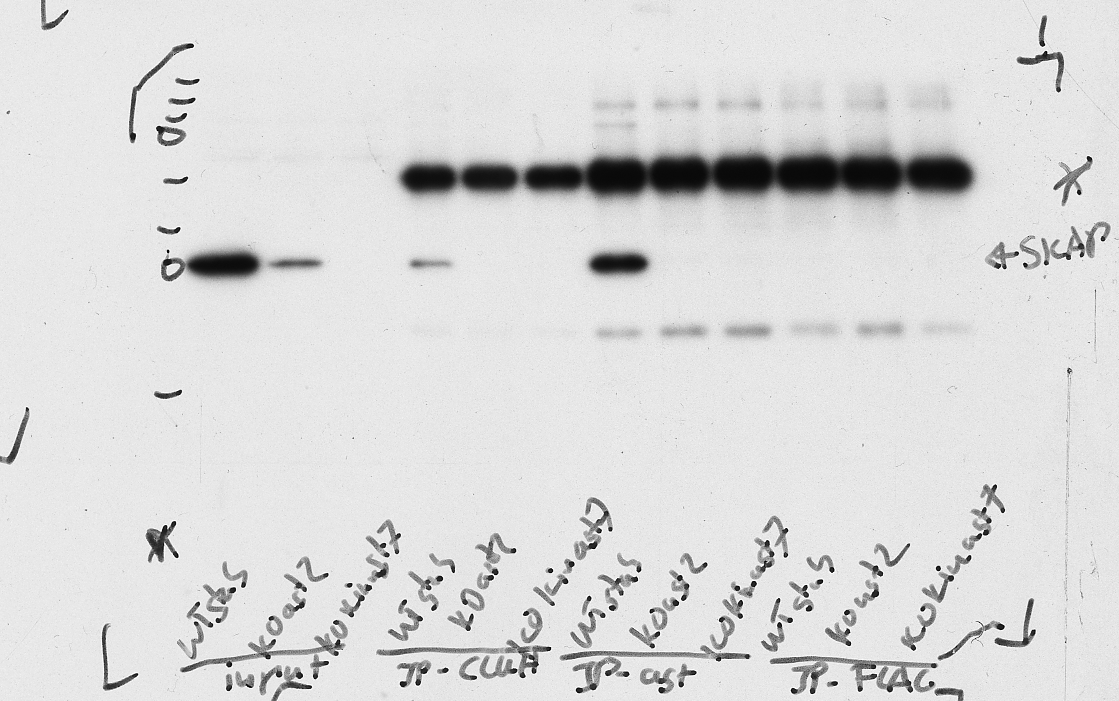

Supplement: Figure 1—figure supplement 1—source data 2. [file elife-74552-fig1-figsupp1-data2.zip › Figure 1-figure supplement 1-source data 2/Uncropped blots for Figure 1-Figure supplement 1D/IP kinastrin.tif]

Figure 2A

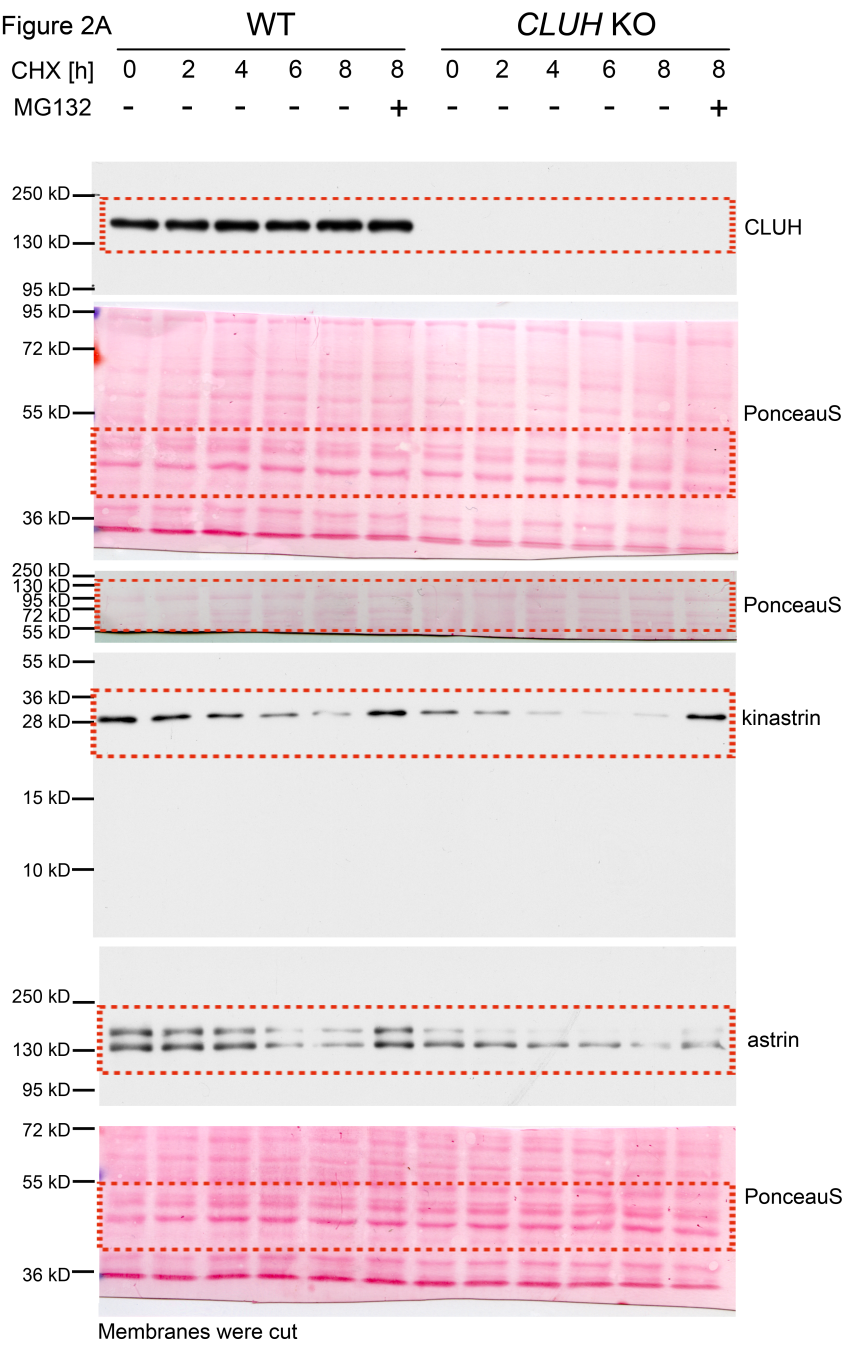

Figure 2H

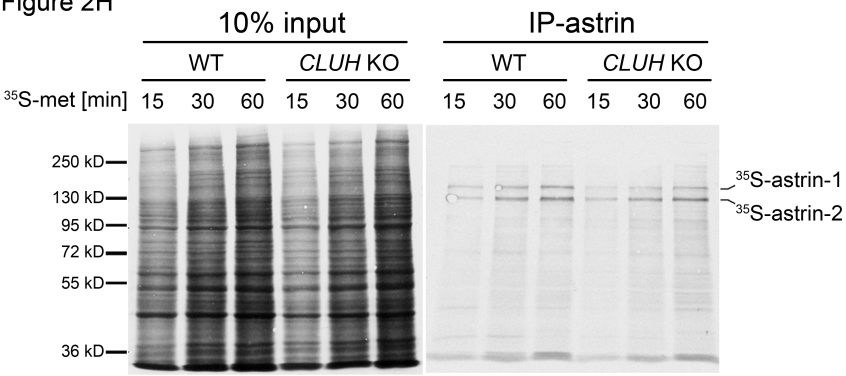

Supplement: Figure 2—source data 1. [file elife-74552-fig2-data1.pdf]

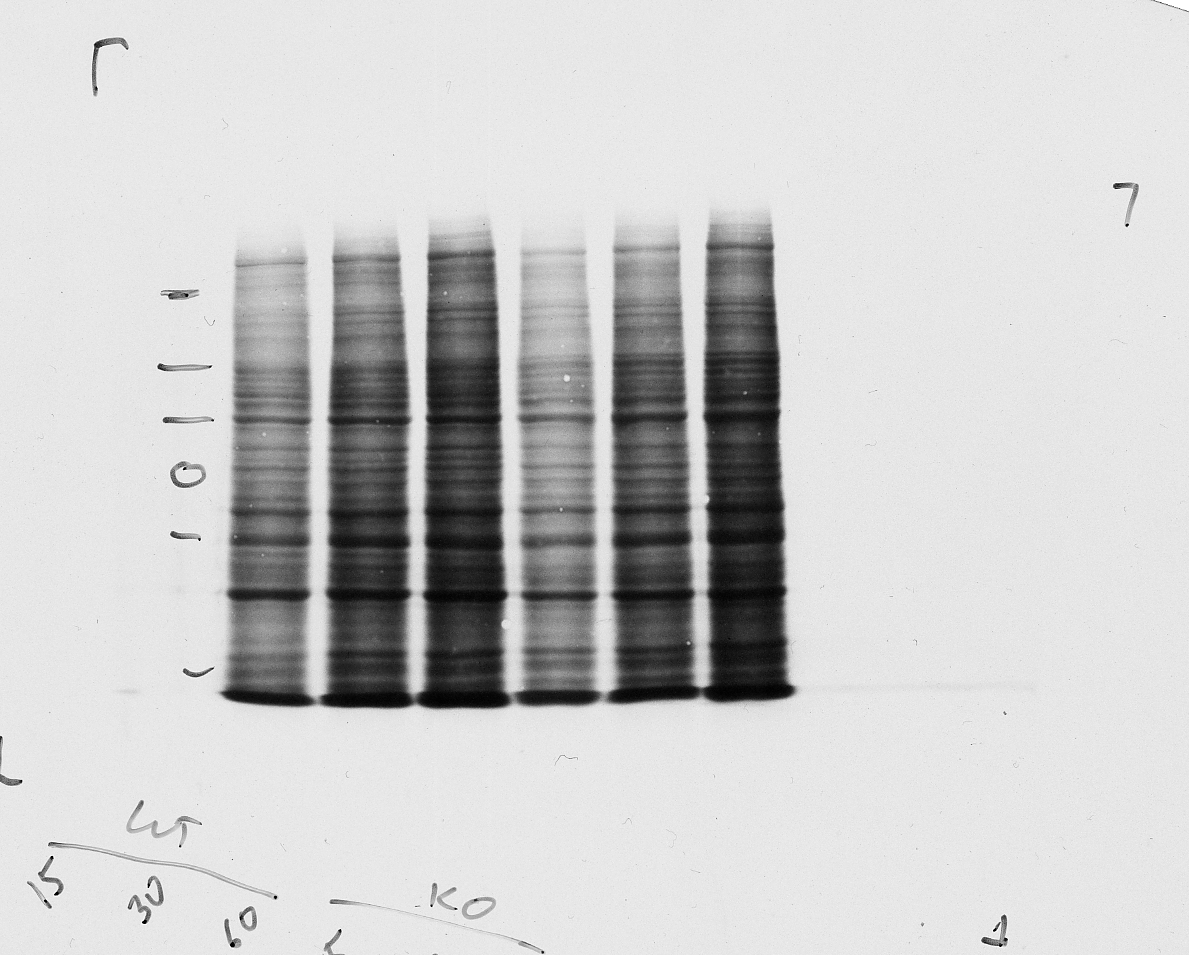

Supplement: Figure 2—source data 2. [file elife-74552-fig2-data2.zip › Figure 2-source data 2/Uncropped blots for Figure 2H/Metabolic labeling Input.tif]

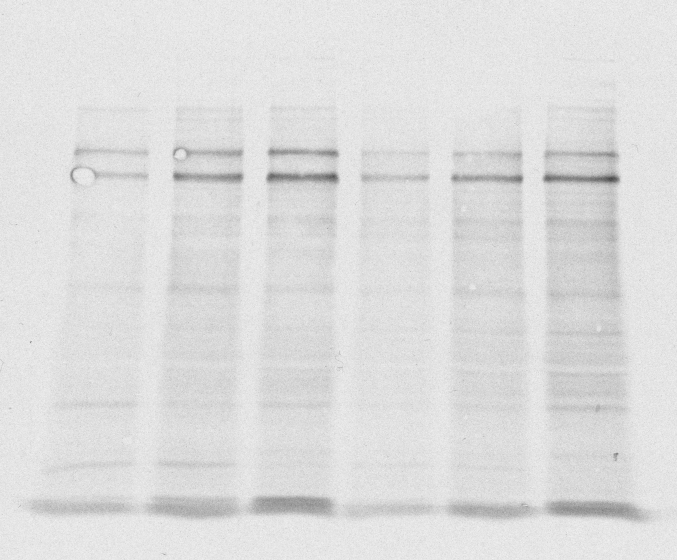

Supplement: Figure 2—source data 2. [file elife-74552-fig2-data2.zip › Figure 2-source data 2/Uncropped blots for Figure 2H/Metabolic labeling IP.tif]

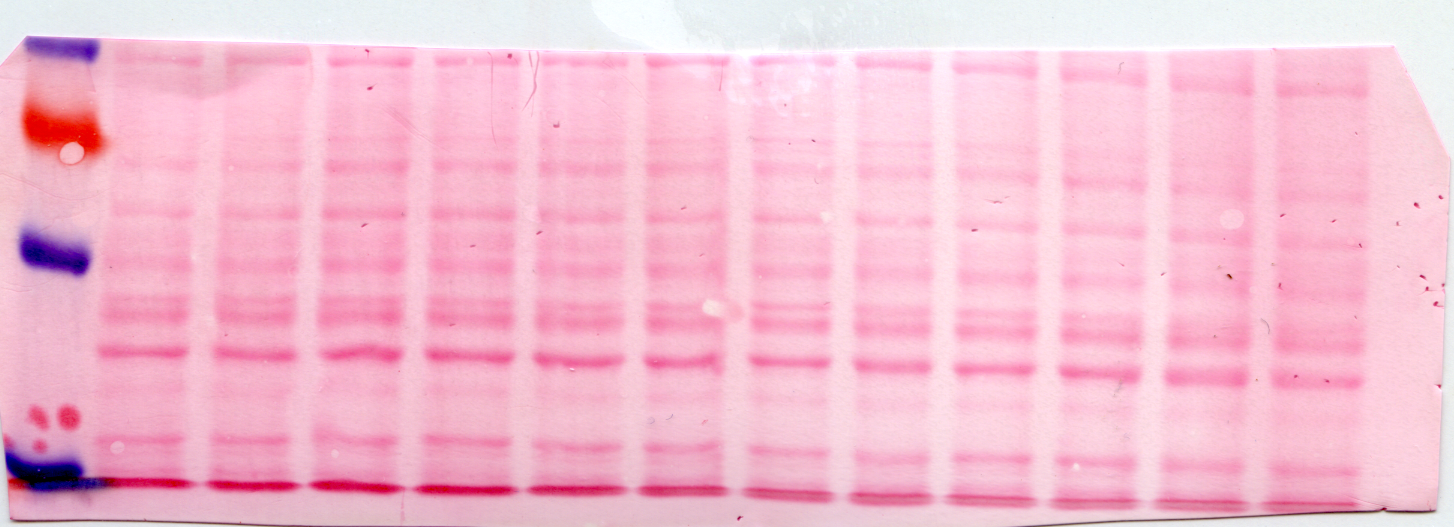

Supplement: Figure 2—source data 2. [file elife-74552-fig2-data2.zip › Figure 2-source data 2/Uncropped blots for Figure 2A/CHX Ponceau S CLUH.tif]

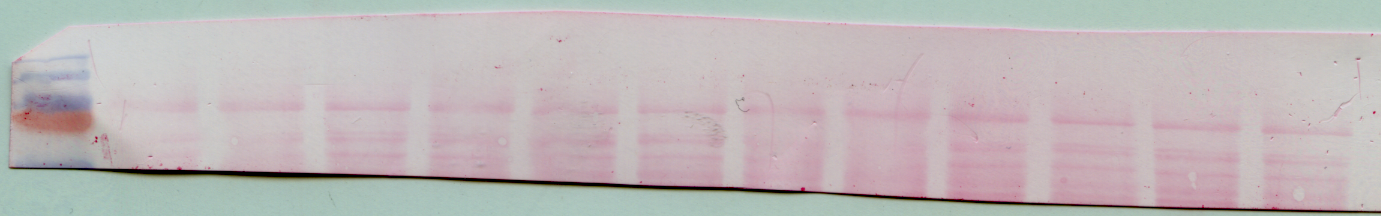

Supplement: Figure 2—source data 2. [file elife-74552-fig2-data2.zip › Figure 2-source data 2/Uncropped blots for Figure 2A/CHX PonceauS kinastrin.tif]

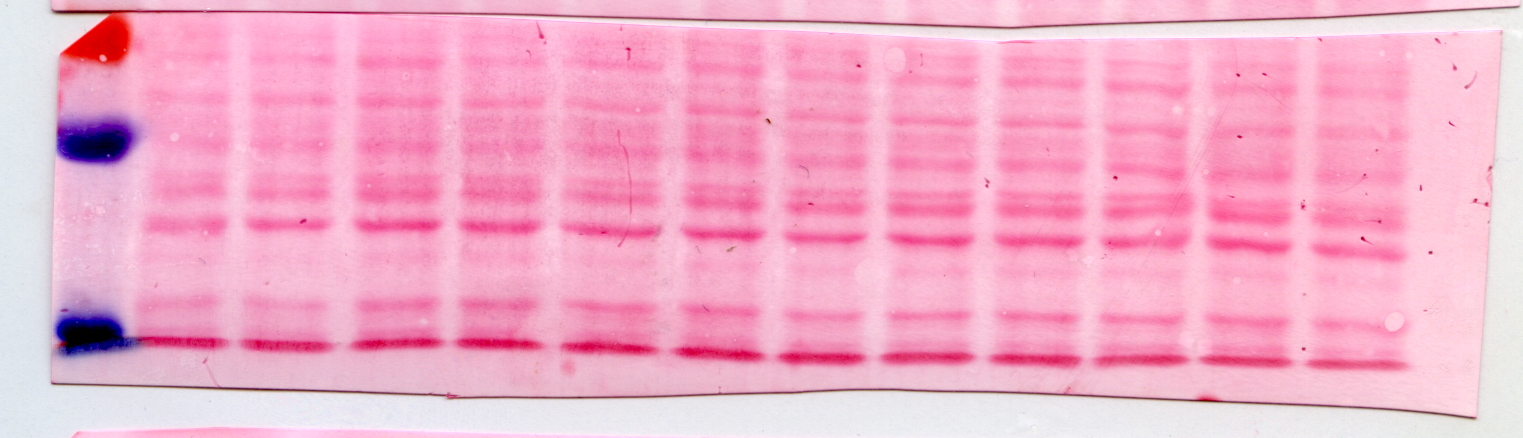

Supplement: Figure 2—source data 2. [file elife-74552-fig2-data2.zip › Figure 2-source data 2/Uncropped blots for Figure 2A/CHX Ponceau S astrin.tif]

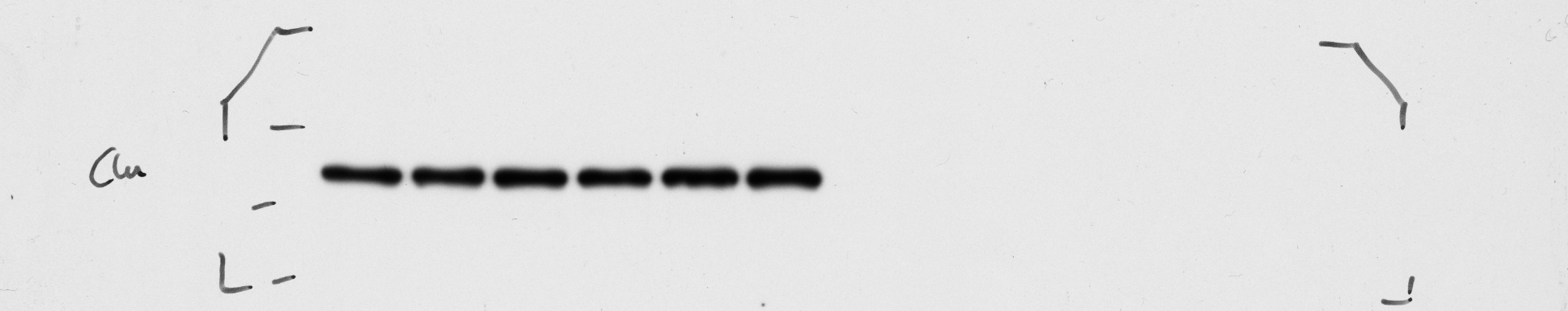

Supplement: Figure 2—source data 2. [file elife-74552-fig2-data2.zip › Figure 2-source data 2/Uncropped blots for Figure 2A/CHX CLUH.tif]

Figure 2-figure supplement 1A

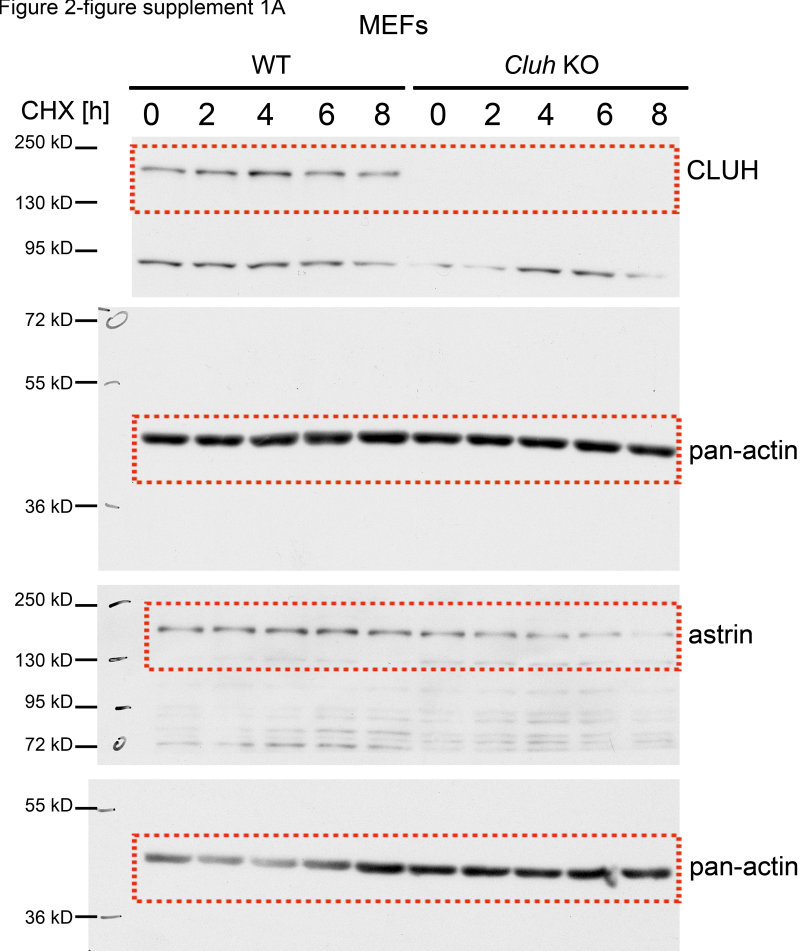

Supplement: Figure 2—figure supplement 1—source data 1. [file elife-74552-fig2-figsupp1-data1.pdf]

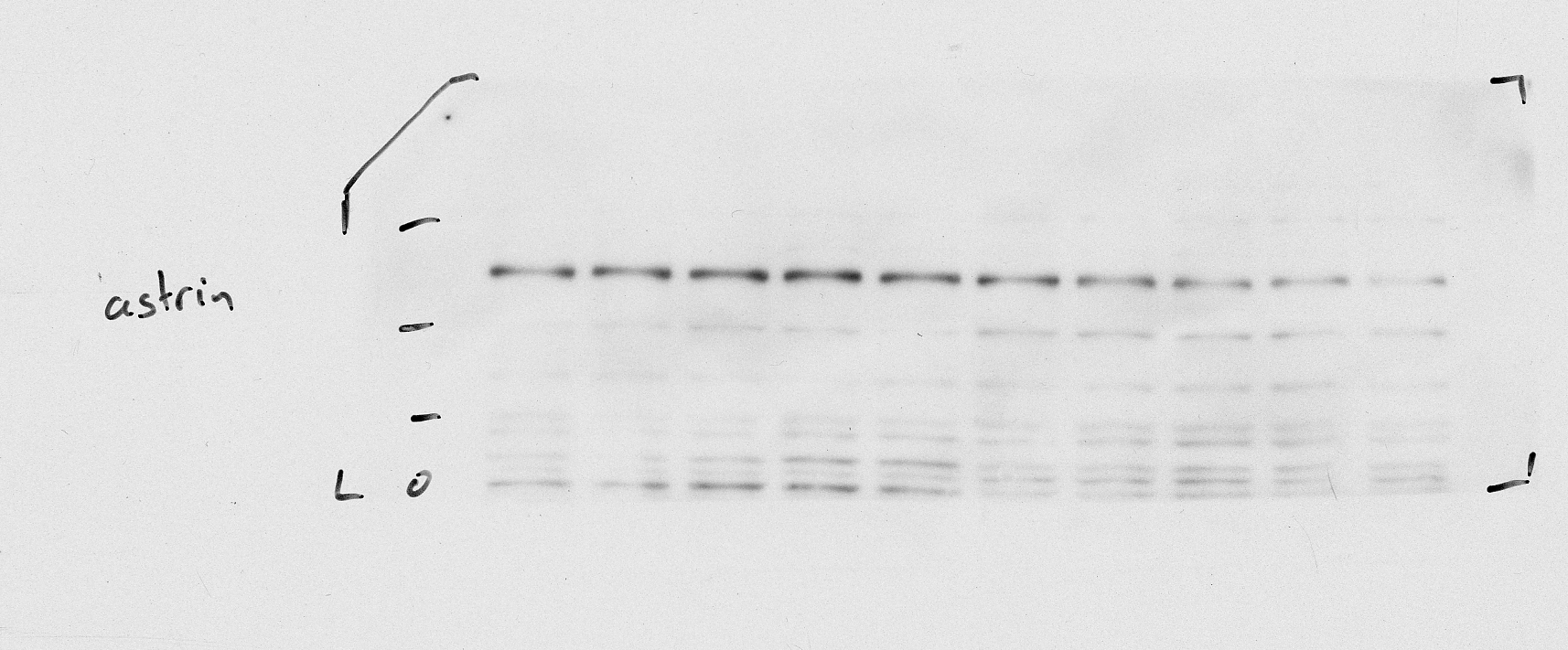

Supplement: Figure 2—figure supplement 1—source data 2. [file elife-74552-fig2-figsupp1-data2.zip › Figure 2-figure supplement 1-source data 2/MEFs astrin.tif]

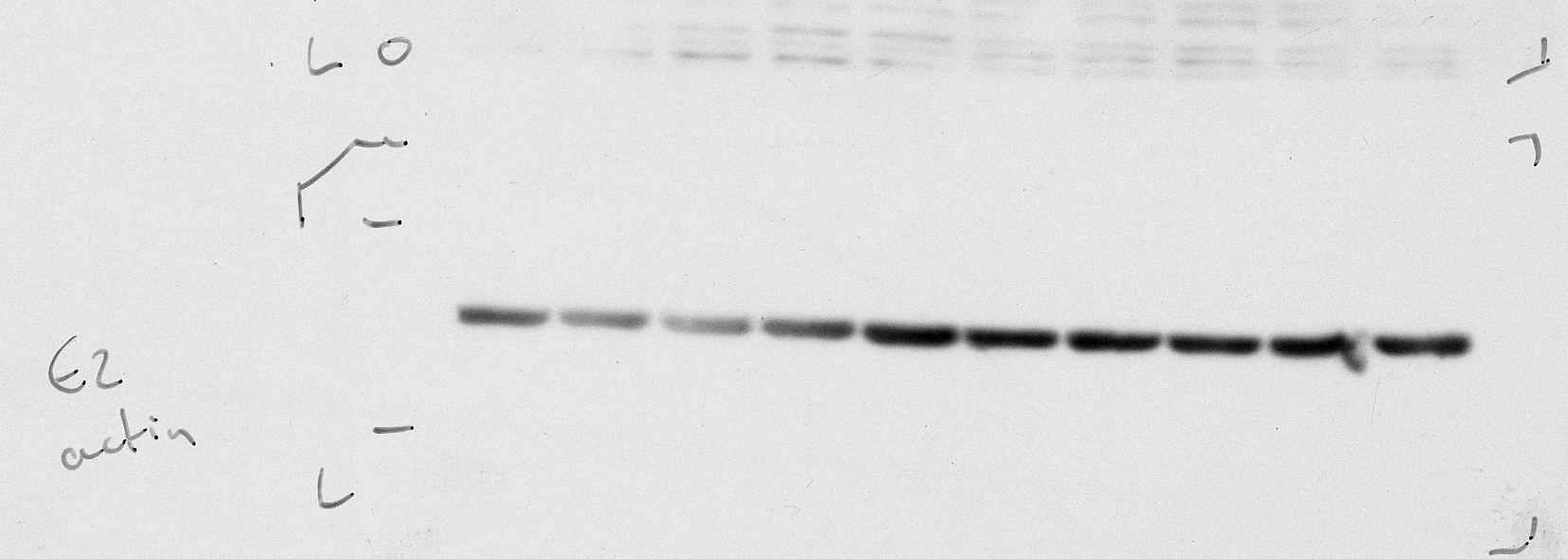

Supplement: Figure 2—figure supplement 1—source data 2. [file elife-74552-fig2-figsupp1-data2.zip › Figure 2-figure supplement 1-source data 2/MEFs astrin actin.tif]

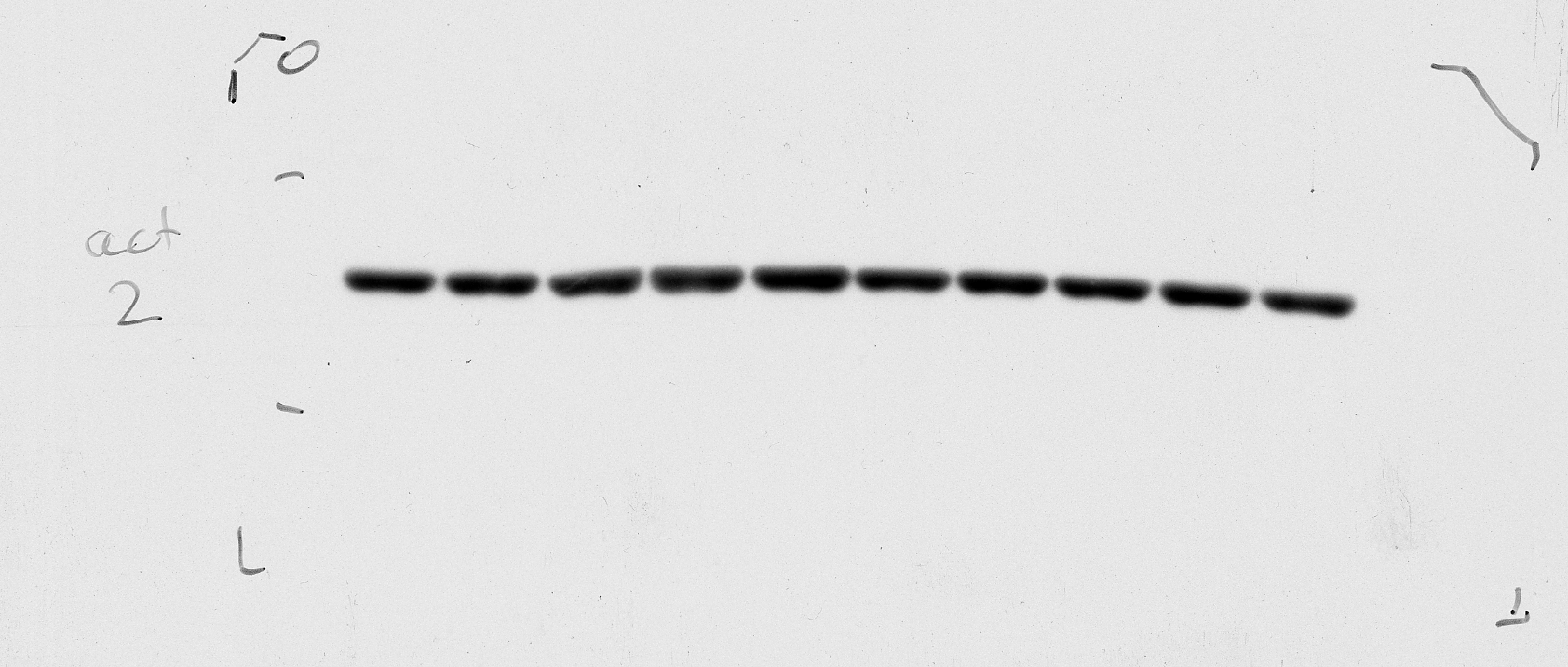

Supplement: Figure 2—figure supplement 1—source data 2. [file elife-74552-fig2-figsupp1-data2.zip › Figure 2-figure supplement 1-source data 2/MEFs CLUH actin.tif]

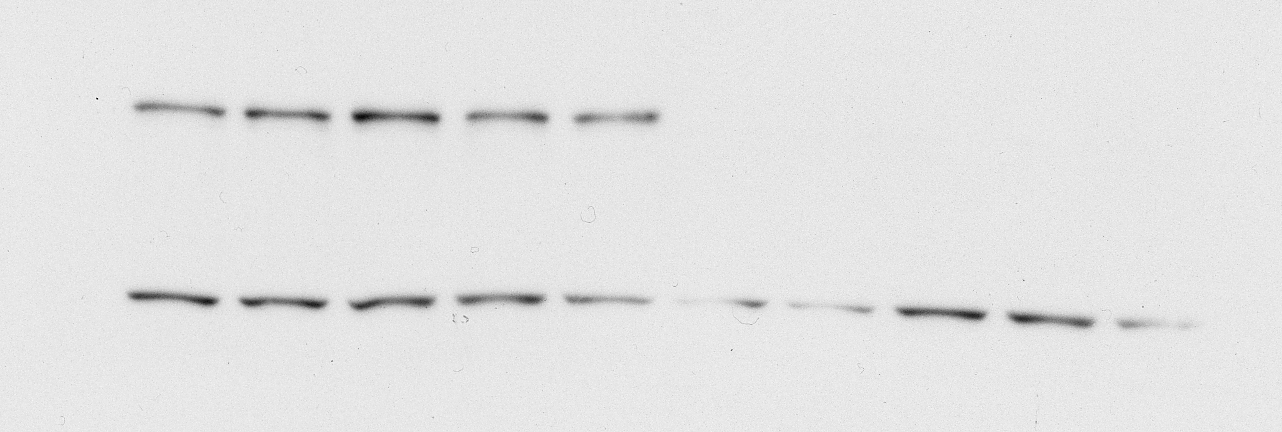

Supplement: Figure 2—figure supplement 1—source data 2. [file elife-74552-fig2-figsupp1-data2.zip › Figure 2-figure supplement 1-source data 2/MEFs CLUH.tif]

Figure 3A

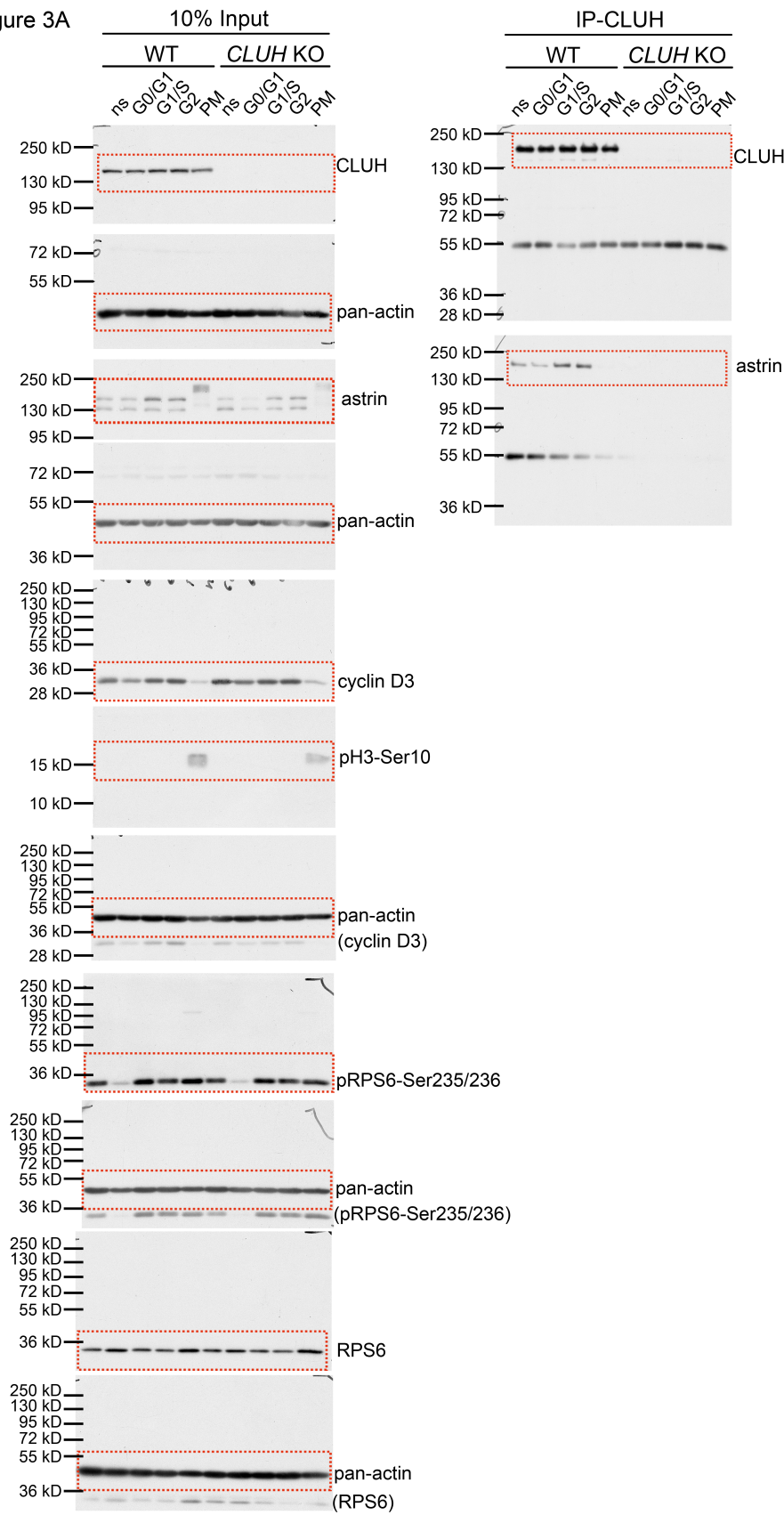

Membranes of input samples were cut

Supplement: Figure 3—source data 1. [file elife-74552-fig3-data1.pdf]

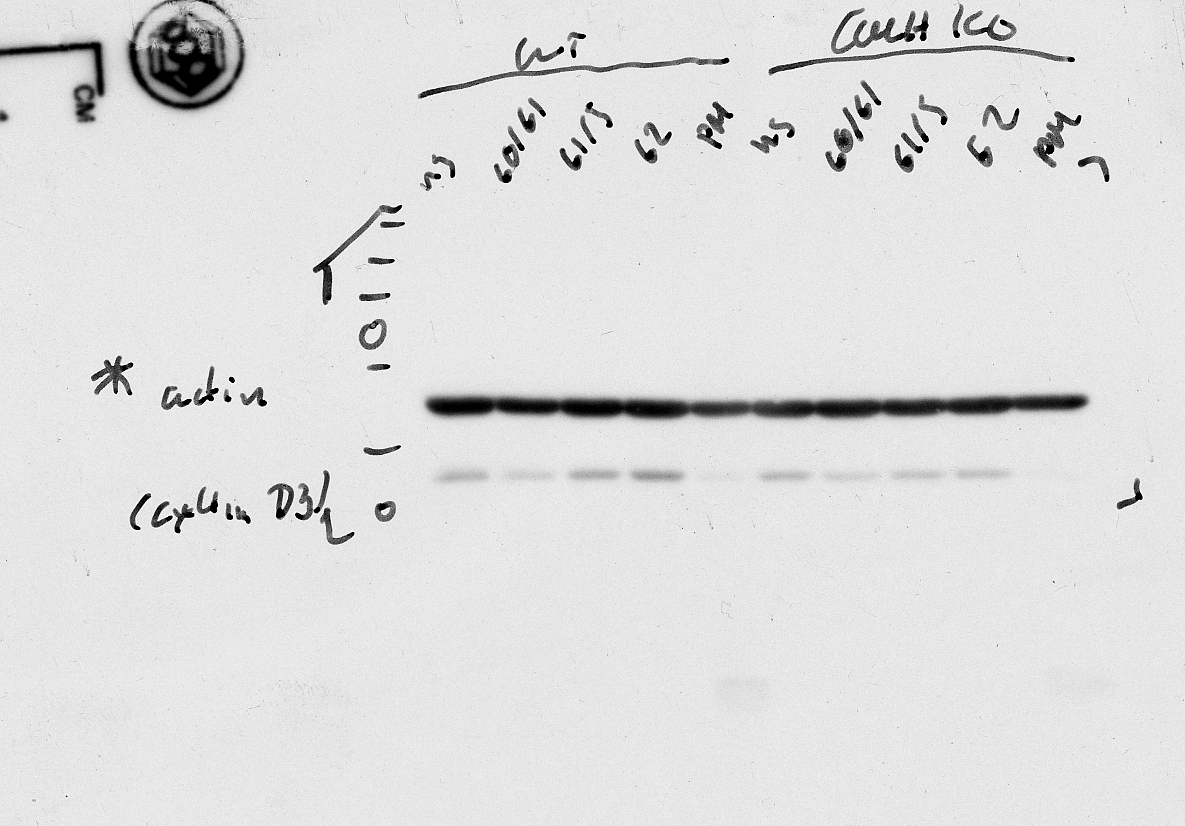

Supplement: Figure 3—source data 2. [file elife-74552-fig3-data2.zip › Figure 3-source data 2/Input cyclin D3 pH3-Ser10 actin.tif]

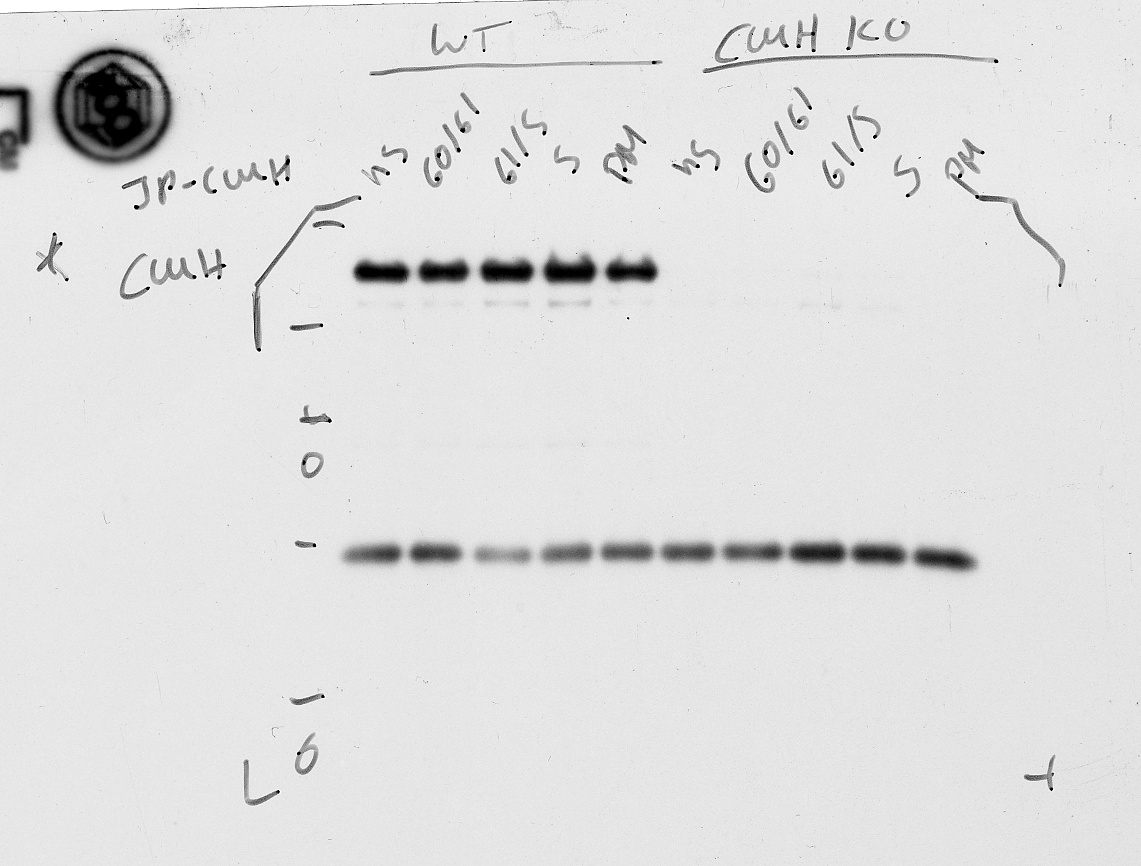

Supplement: Figure 3—source data 2. [file elife-74552-fig3-data2.zip › Figure 3-source data 2/IP CLUH.tif]

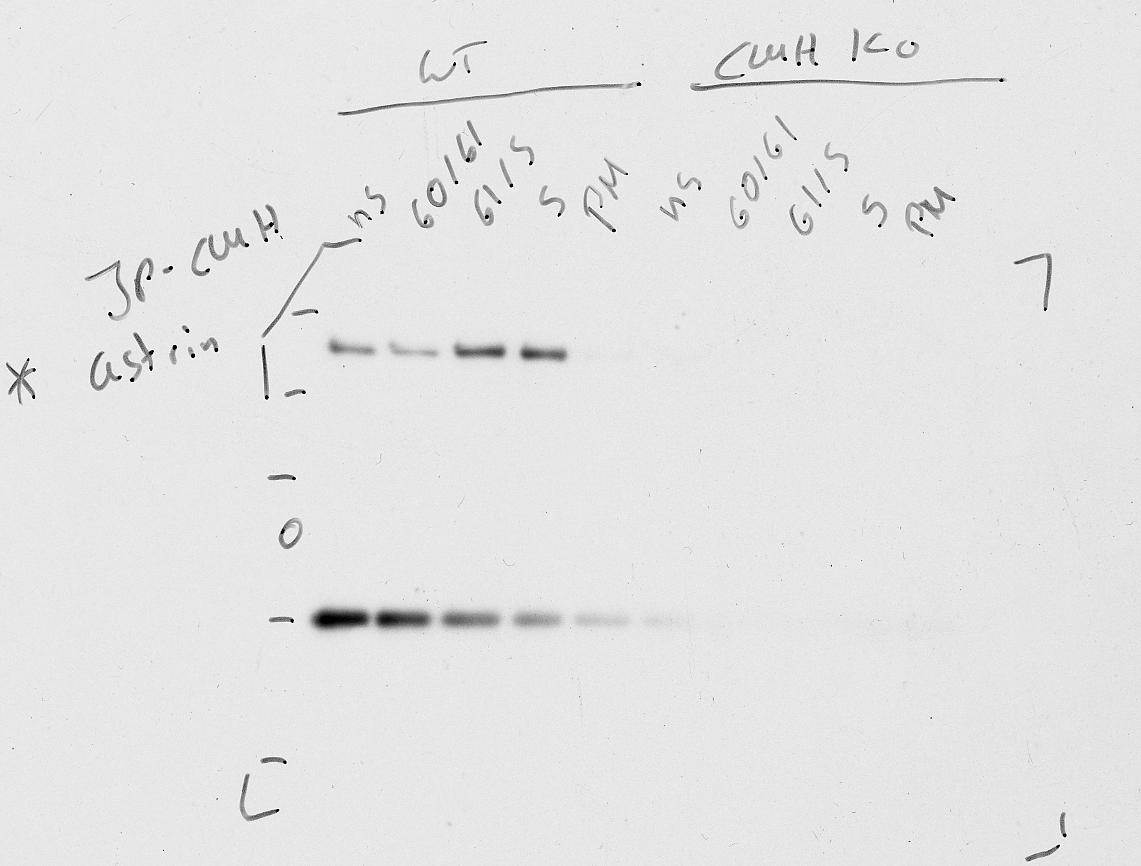

Supplement: Figure 3—source data 2. [file elife-74552-fig3-data2.zip › Figure 3-source data 2/IP astrin.tif]

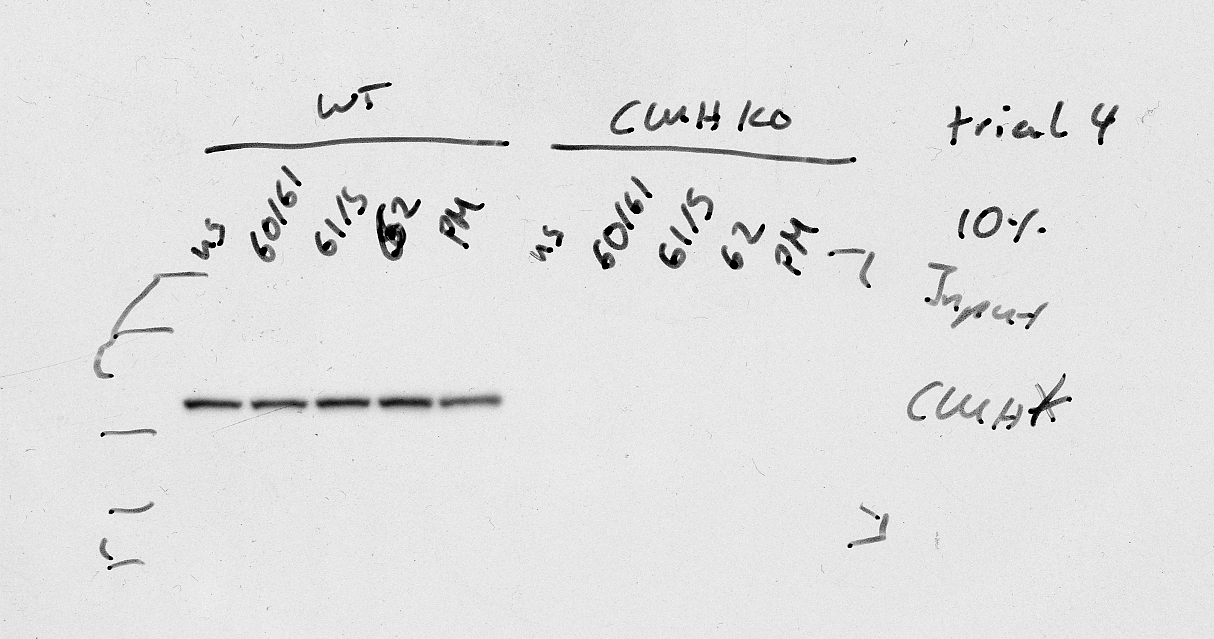

Supplement: Figure 3—source data 2. [file elife-74552-fig3-data2.zip › Figure 3-source data 2/Input CLUH.tif]

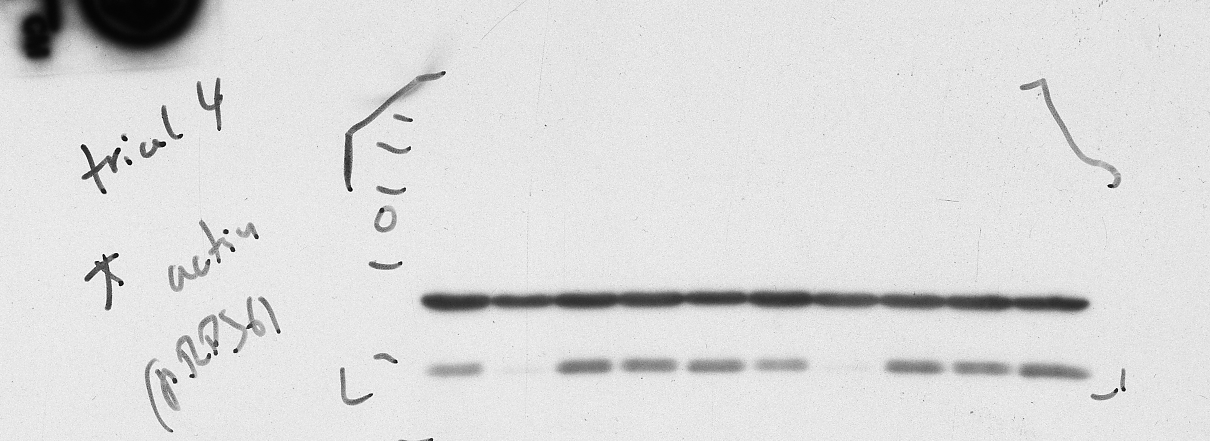

Supplement: Figure 3—source data 2. [file elife-74552-fig3-data2.zip › Figure 3-source data 2/Input pRPS6-Ser235236 actin.tif]

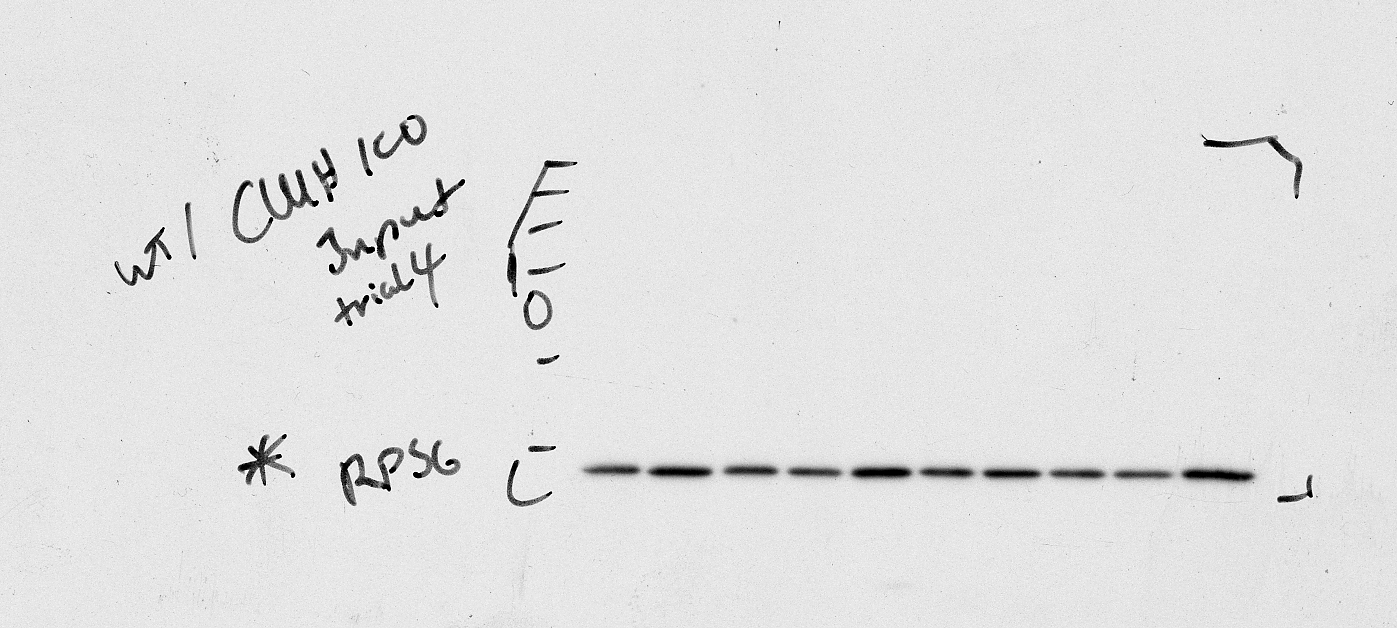

Supplement: Figure 3—source data 2. [file elife-74552-fig3-data2.zip › Figure 3-source data 2/Input RPS6.tif]

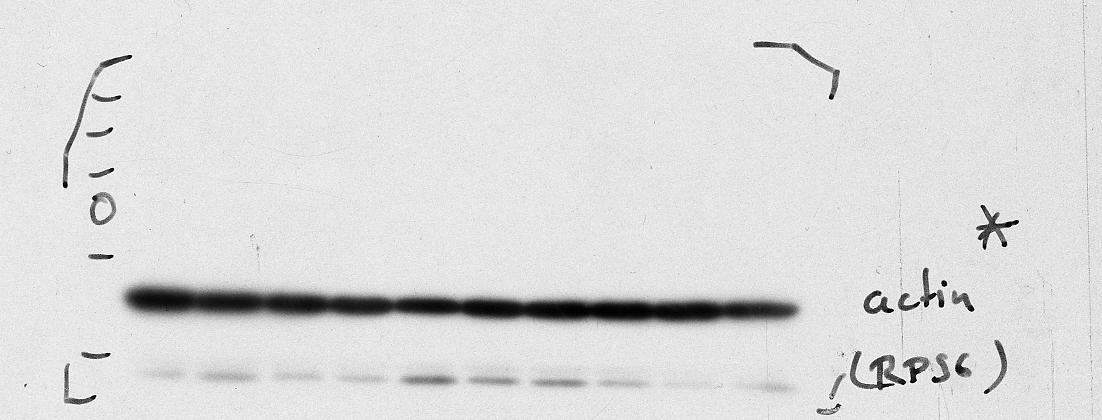

Supplement: Figure 3—source data 2. [file elife-74552-fig3-data2.zip › Figure 3-source data 2/Input RPS6 actin.tif]

Figure 4D

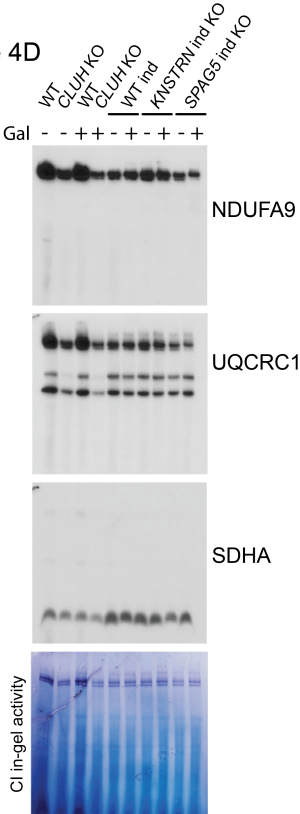

Supplement: Figure 4—source data 1. [file elife-74552-fig4-data1.pdf]

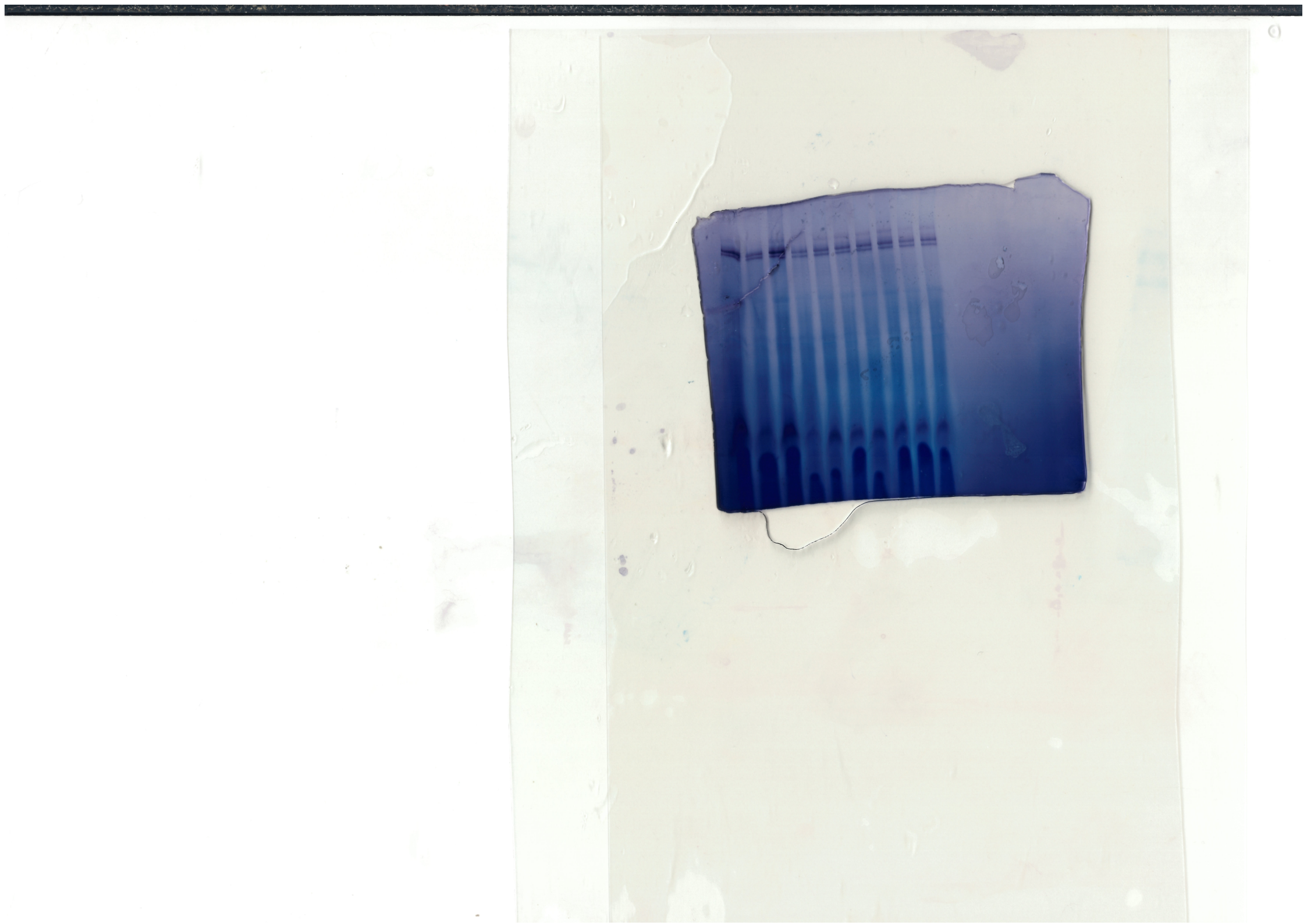

Supplement: Figure 4—source data 2. [file elife-74552-fig4-data2.zip › Figure 4-source data 2/CI in gel activity.pdf]

10.09.11 - US

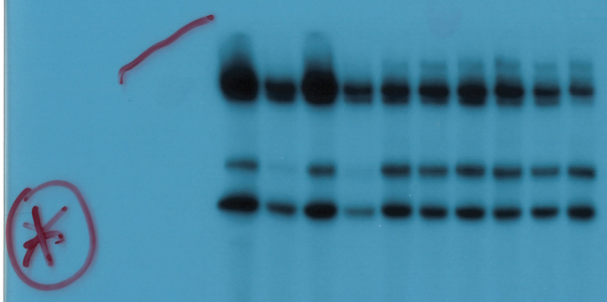

CIII 49000

SC  
or AJ

BNE 49000 RLA 12000  
SC

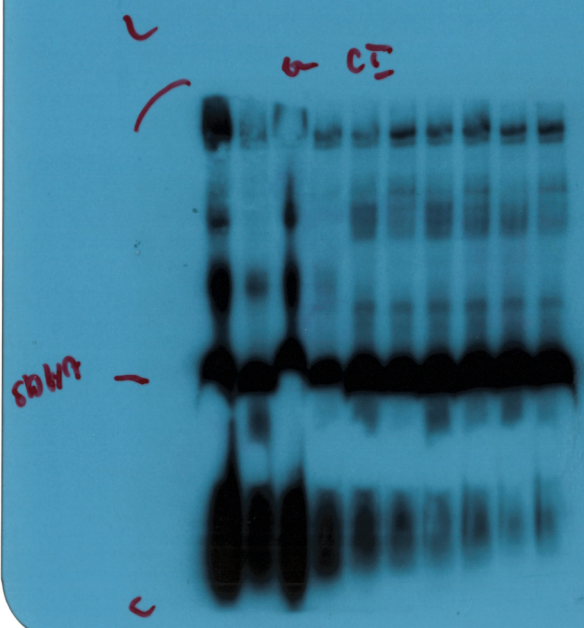

or CII

49000

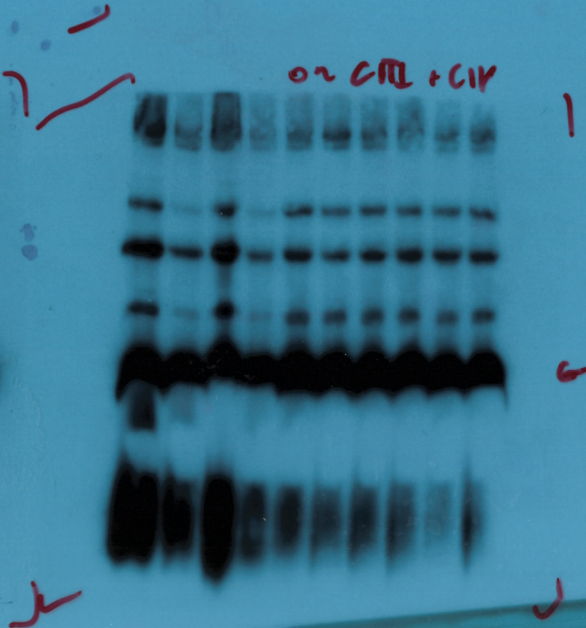

or CIII + CII

49000

Supplement: Figure 4—source data 2. [file elife-74552-fig4-data2.zip › Figure 4-source data 2/UQCRC1.pdf]

A

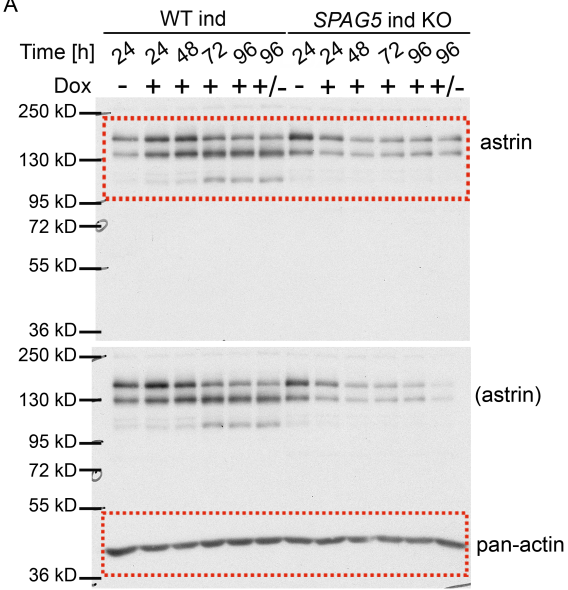

B

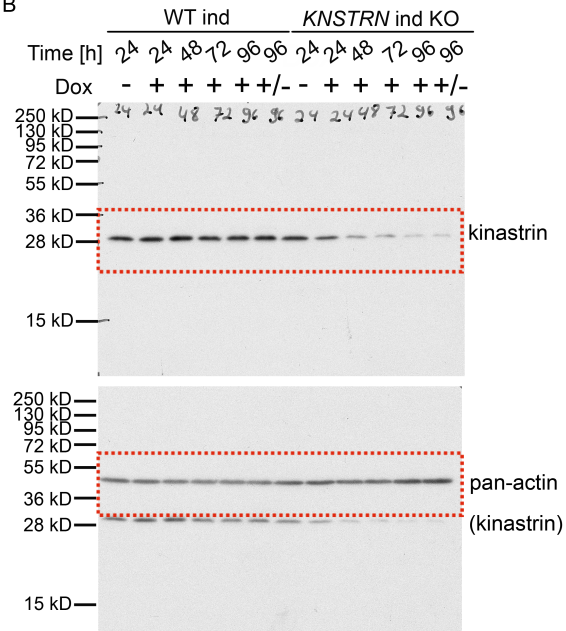

Supplement: Figure 4—figure supplement 1—source data 1. [file elife-74552-fig4-figsupp1-data1.pdf]

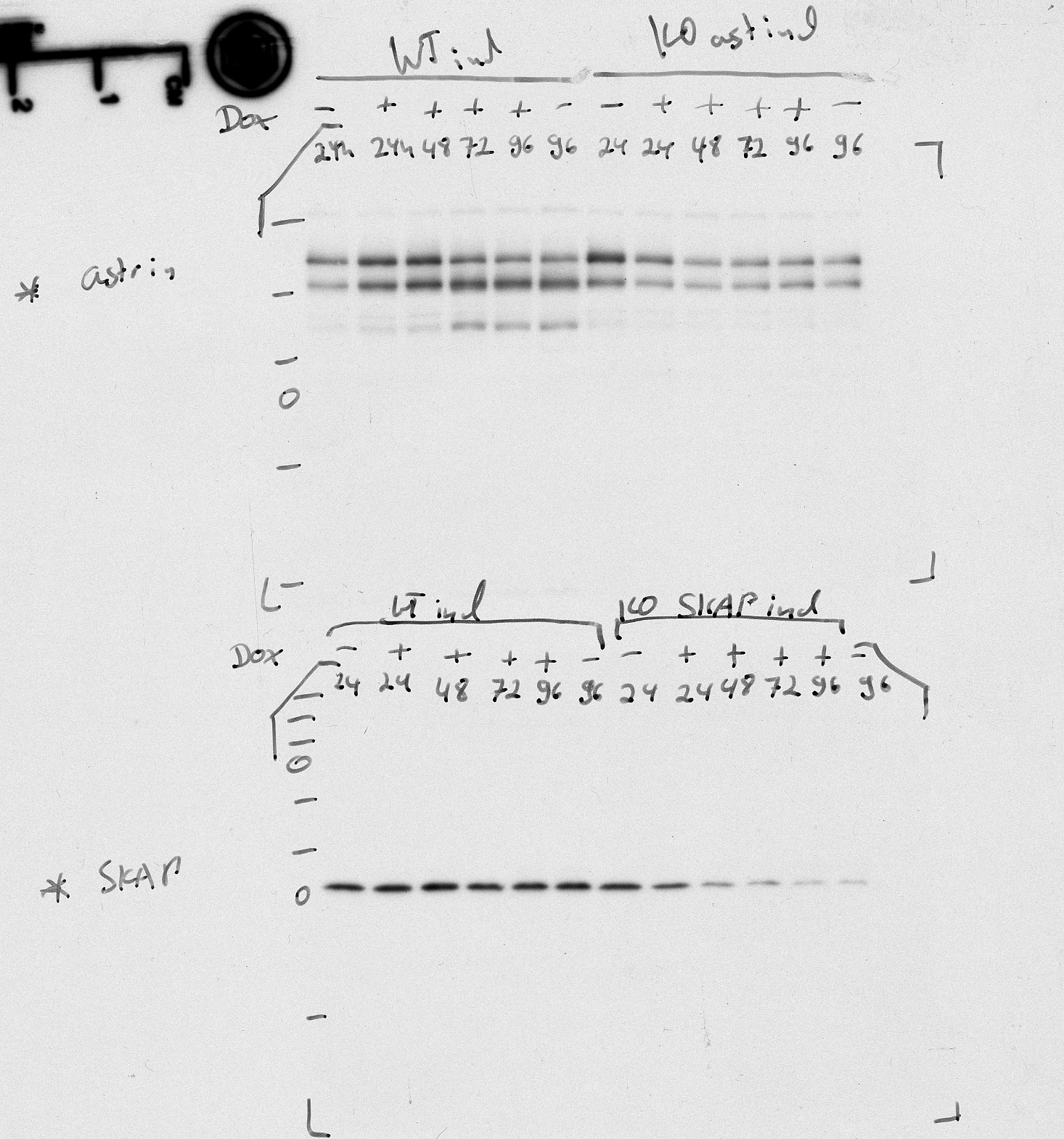

Supplement: Figure 4—figure supplement 1—source data 2. [file elife-74552-fig4-figsupp1-data2.zip › Figure 4-figure supplement 1-source data 2/A and B astrin kinastrin.tif]

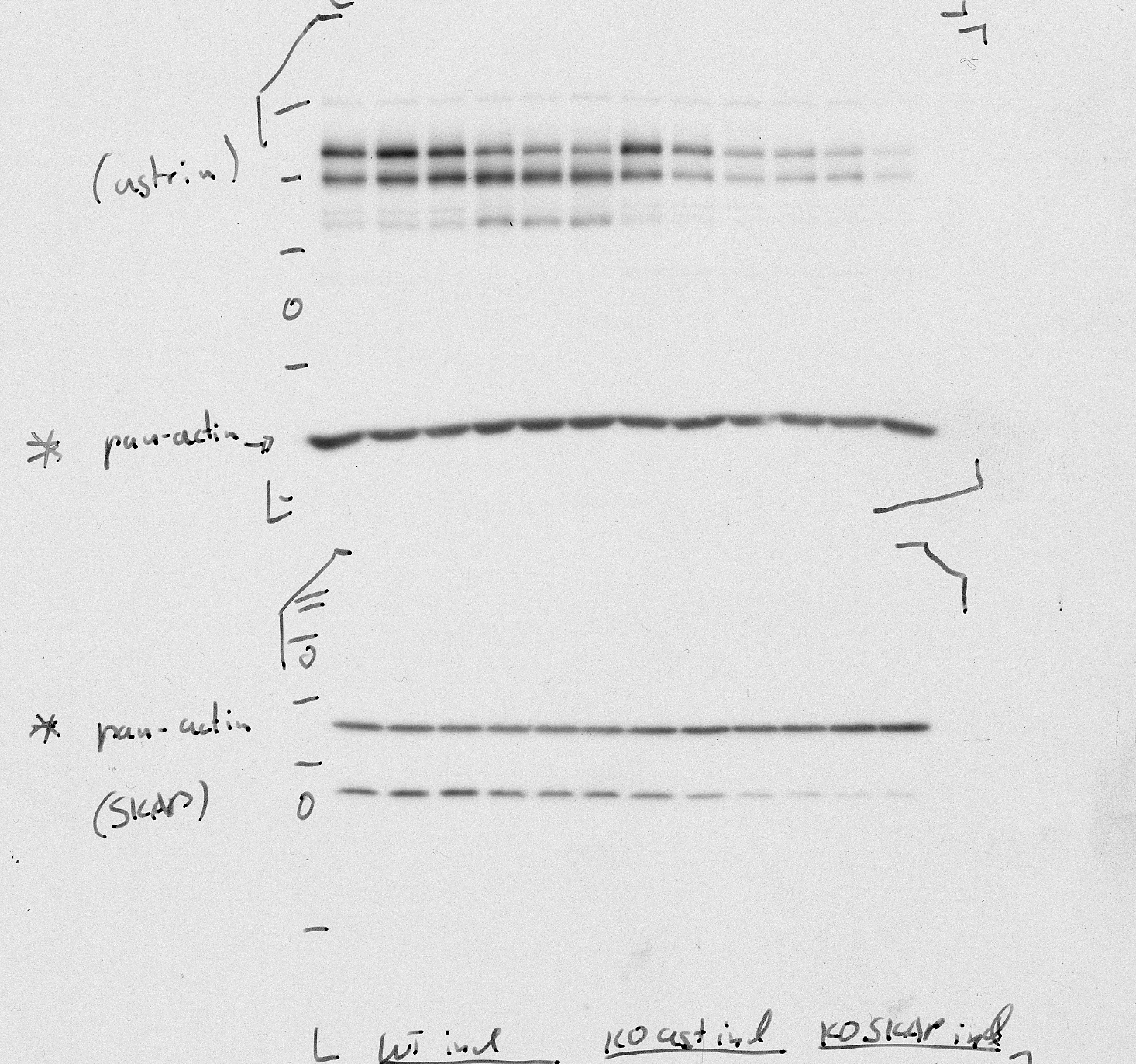

Supplement: Figure 4—figure supplement 1—source data 2. [file elife-74552-fig4-figsupp1-data2.zip › Figure 4-figure supplement 1-source data 2/A and B pan-actin.tif]

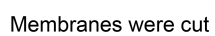

Membranes were cut

Supplement: Figure 5—figure supplement 1—source data 1. [file elife-74552-fig5-figsupp1-data1.pdf]

Figure 7A

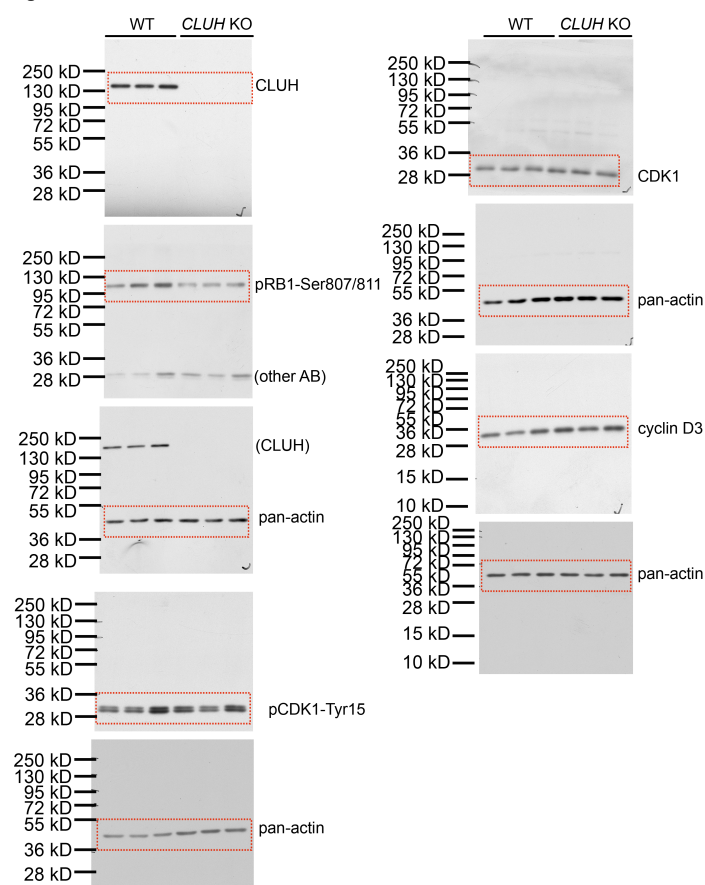

Figure 7H

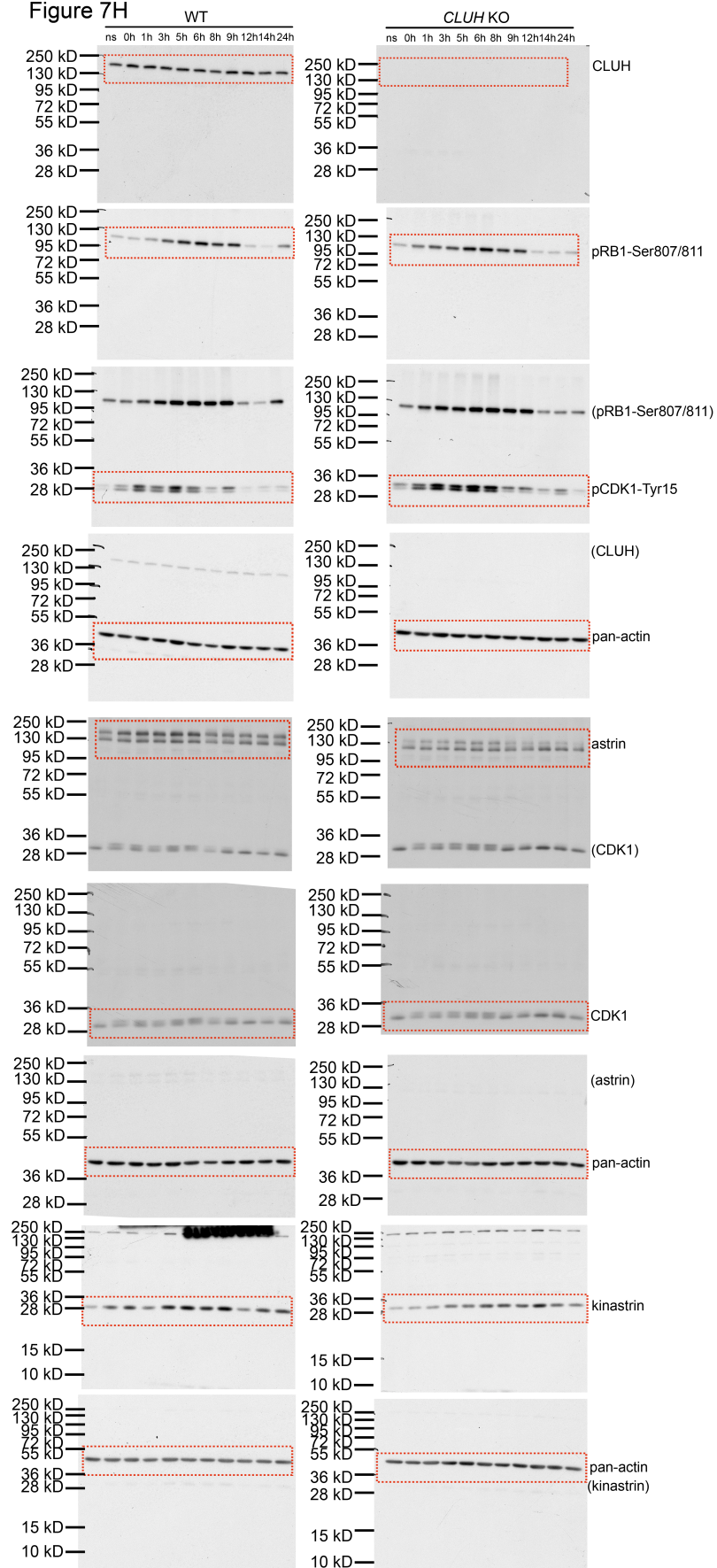

Figure 7H

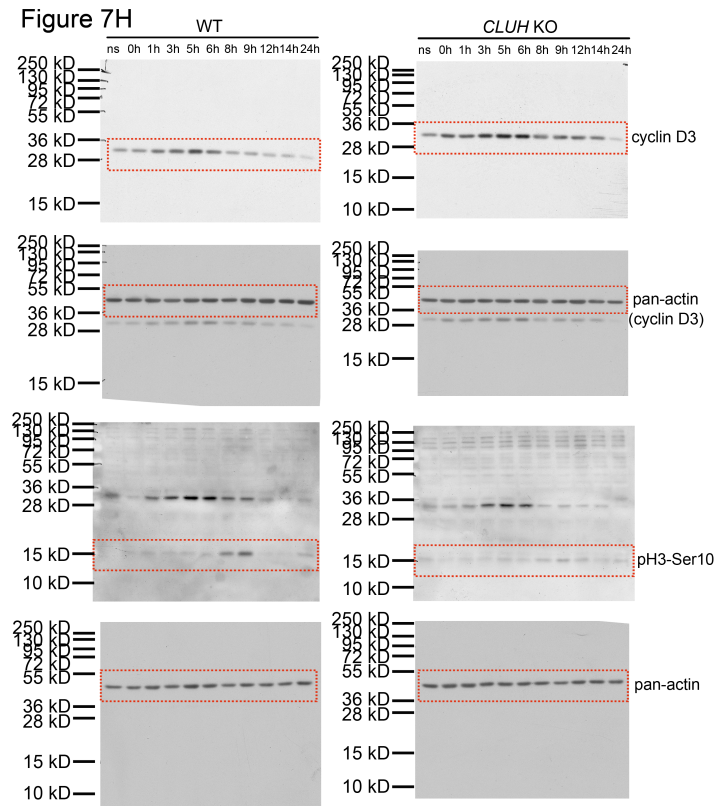

Supplement: Figure 7—source data 1. [file elife-74552-fig7-data1.pdf]

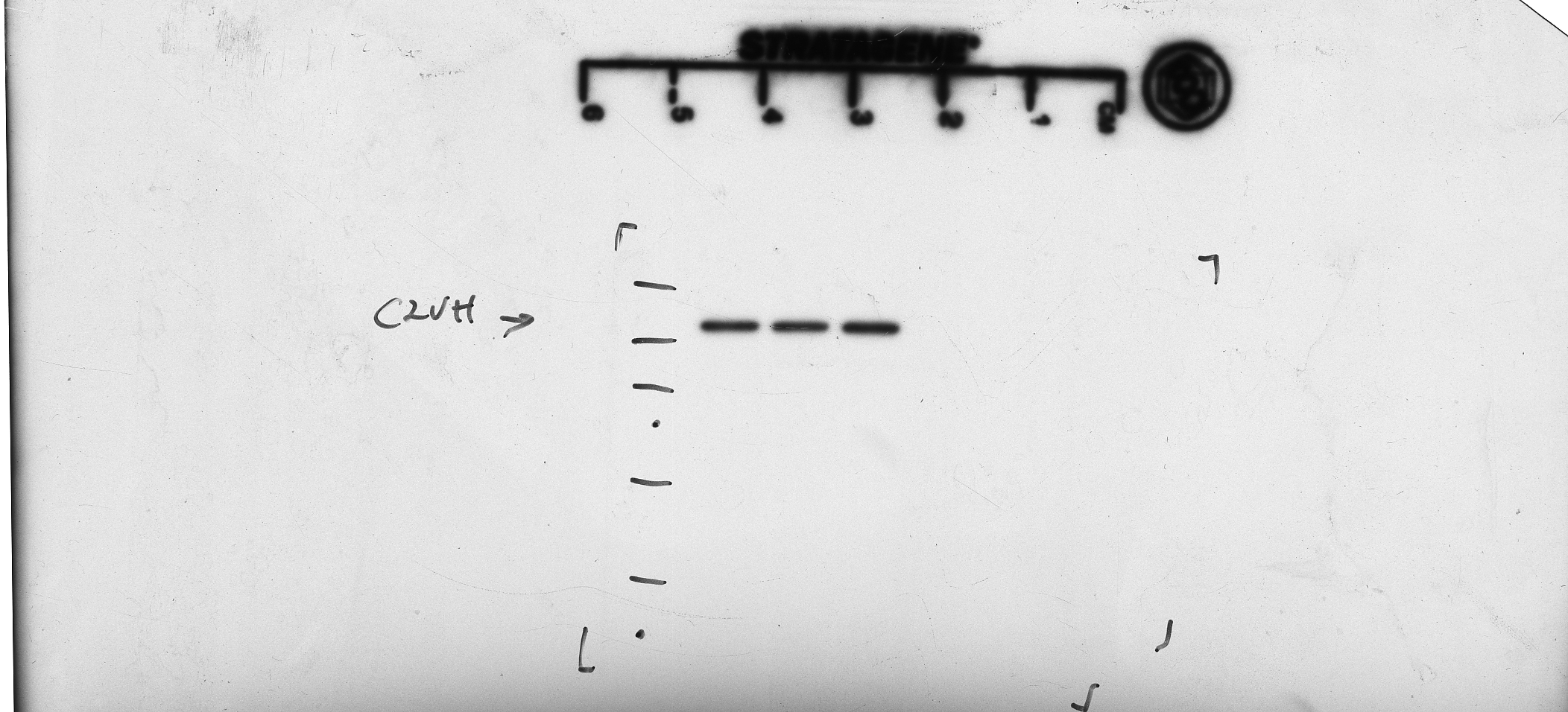

Supplement: Figure 7—source data 2. [file elife-74552-fig7-data2.zip › Figure 7ΓÇösource data 2/Uncropped blots for Figure 7A/CLUH019.tif]

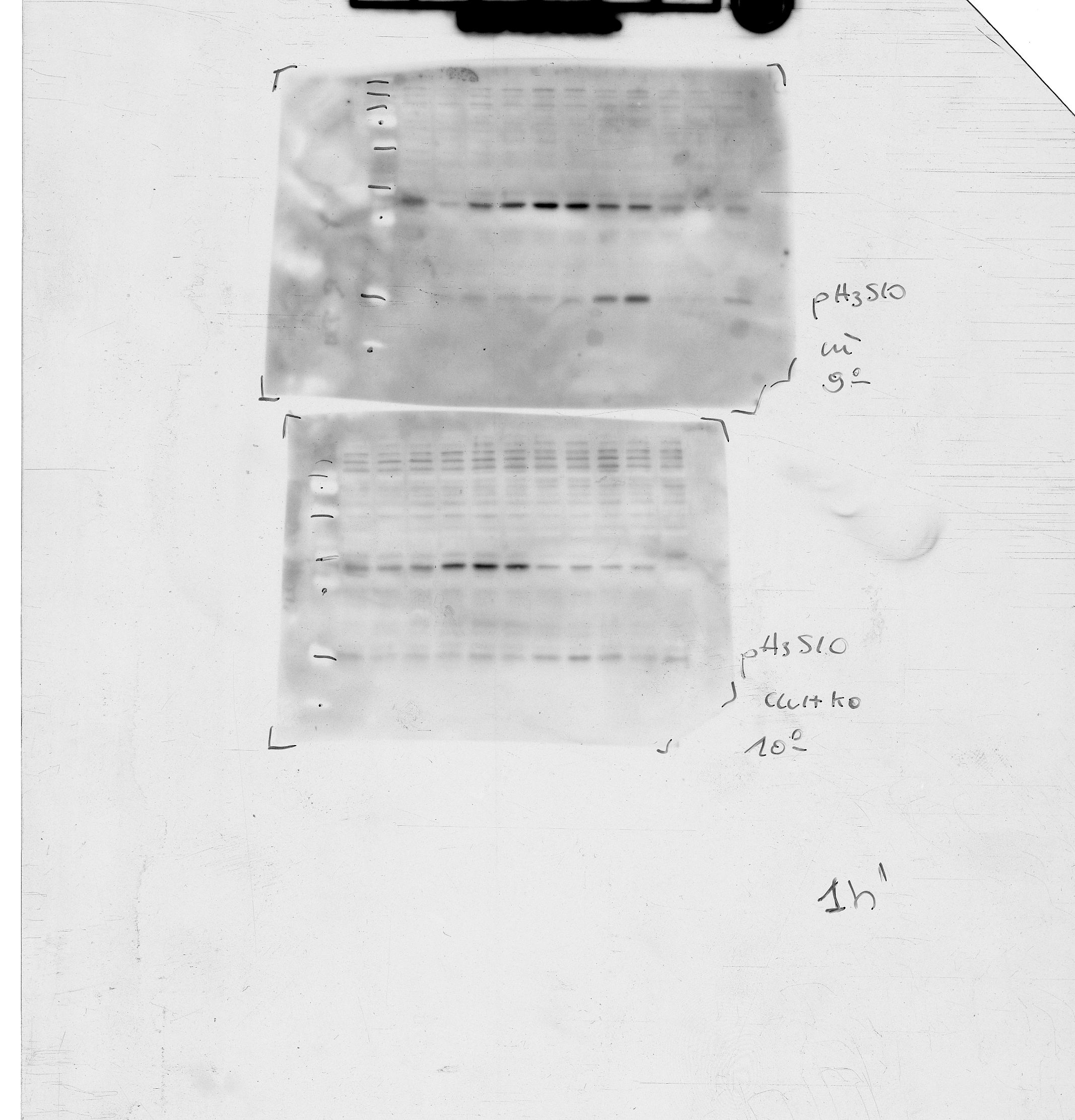

Supplement: Figure 7—source data 2. [file elife-74552-fig7-data2.zip › Figure 7ΓÇösource data 2/Uncropped blots for Figure 7H/pH3-Ser10.tif]

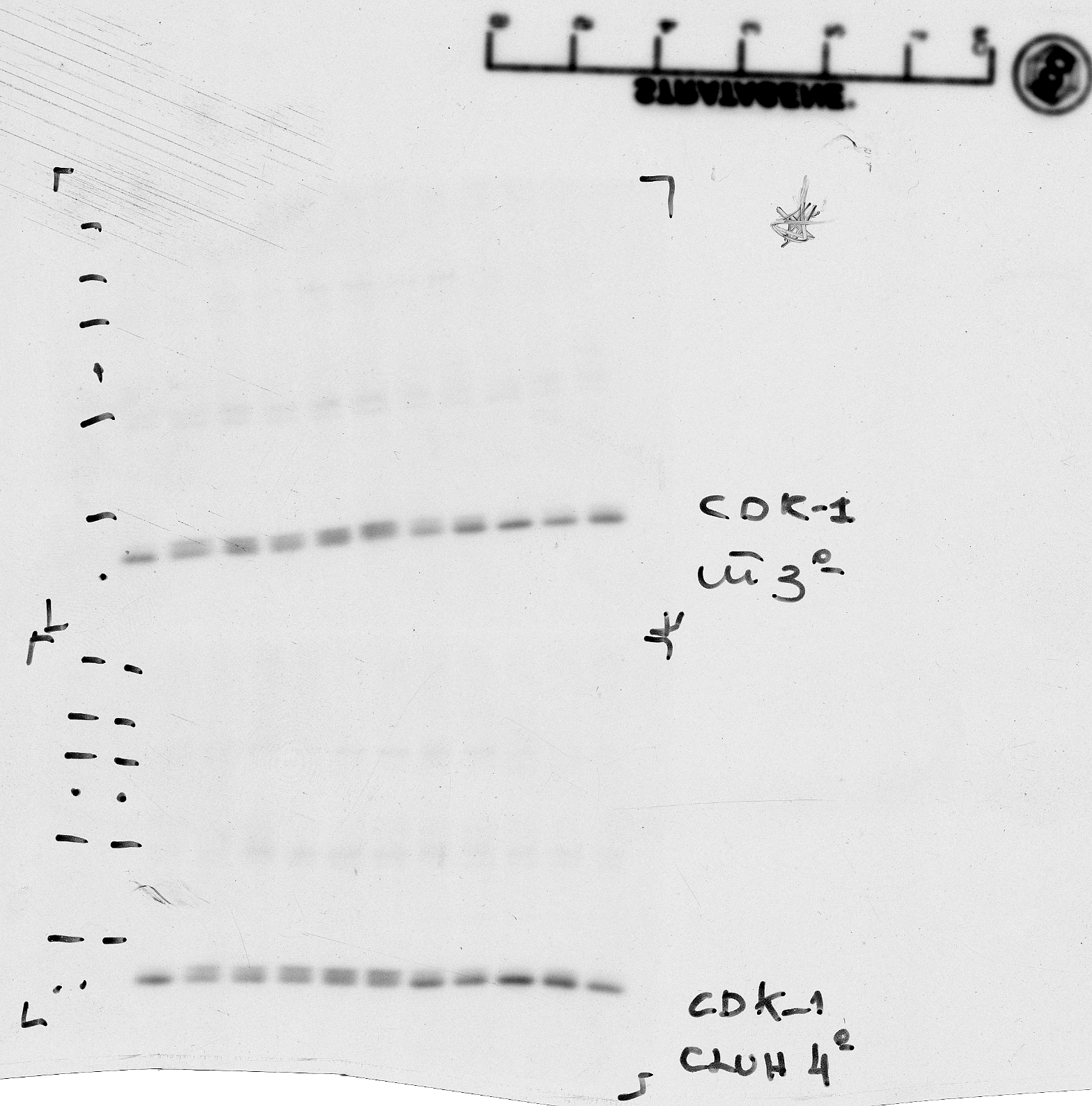

Supplement: Figure 7—source data 2. [file elife-74552-fig7-data2.zip › Figure 7ΓÇösource data 2/Uncropped blots for Figure 7H/CDK1.tif]

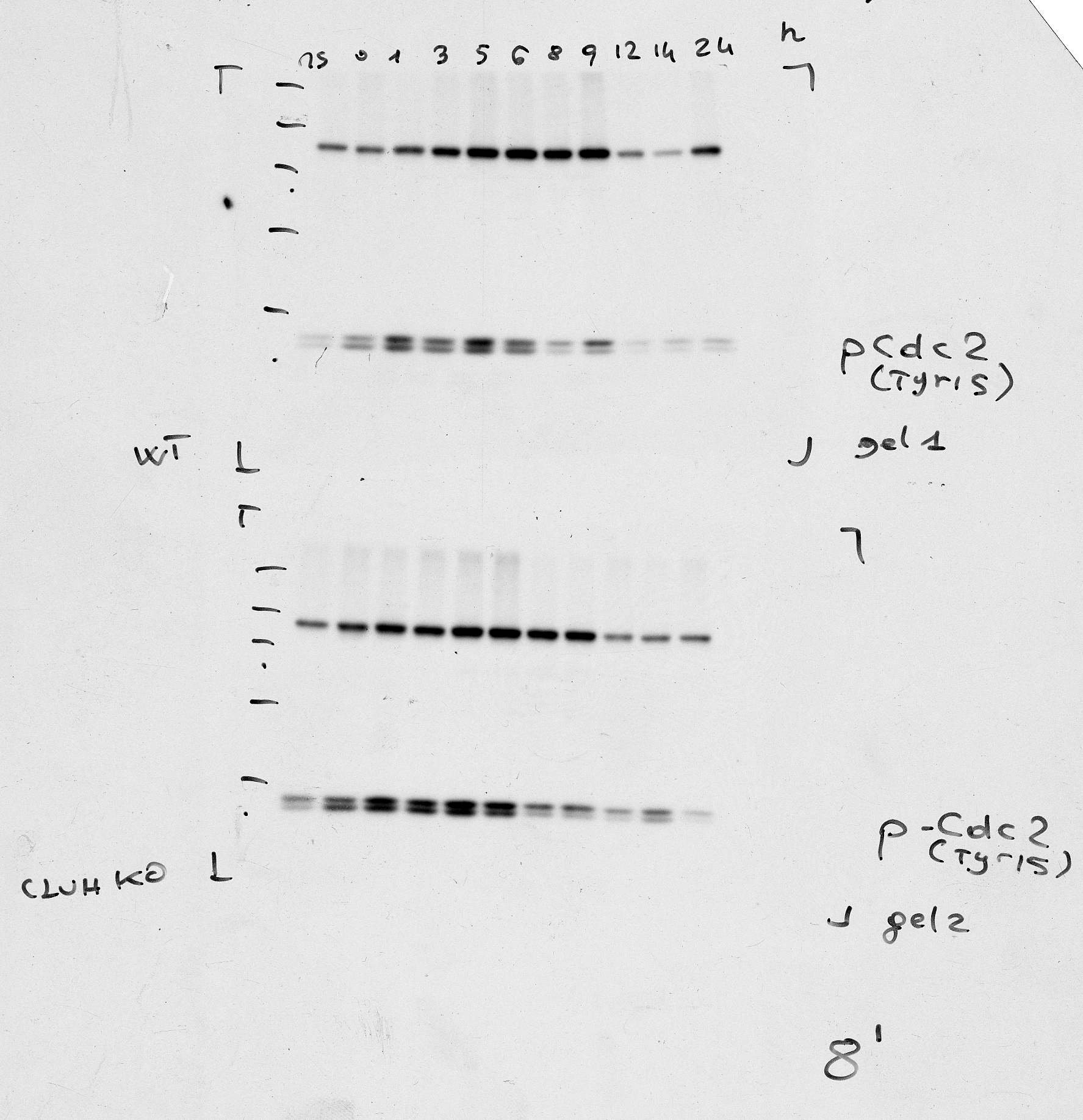

Supplement: Figure 7—source data 2. [file elife-74552-fig7-data2.zip › Figure 7ΓÇösource data 2/Uncropped blots for Figure 7H/pCDK1-Tyr15.tif]

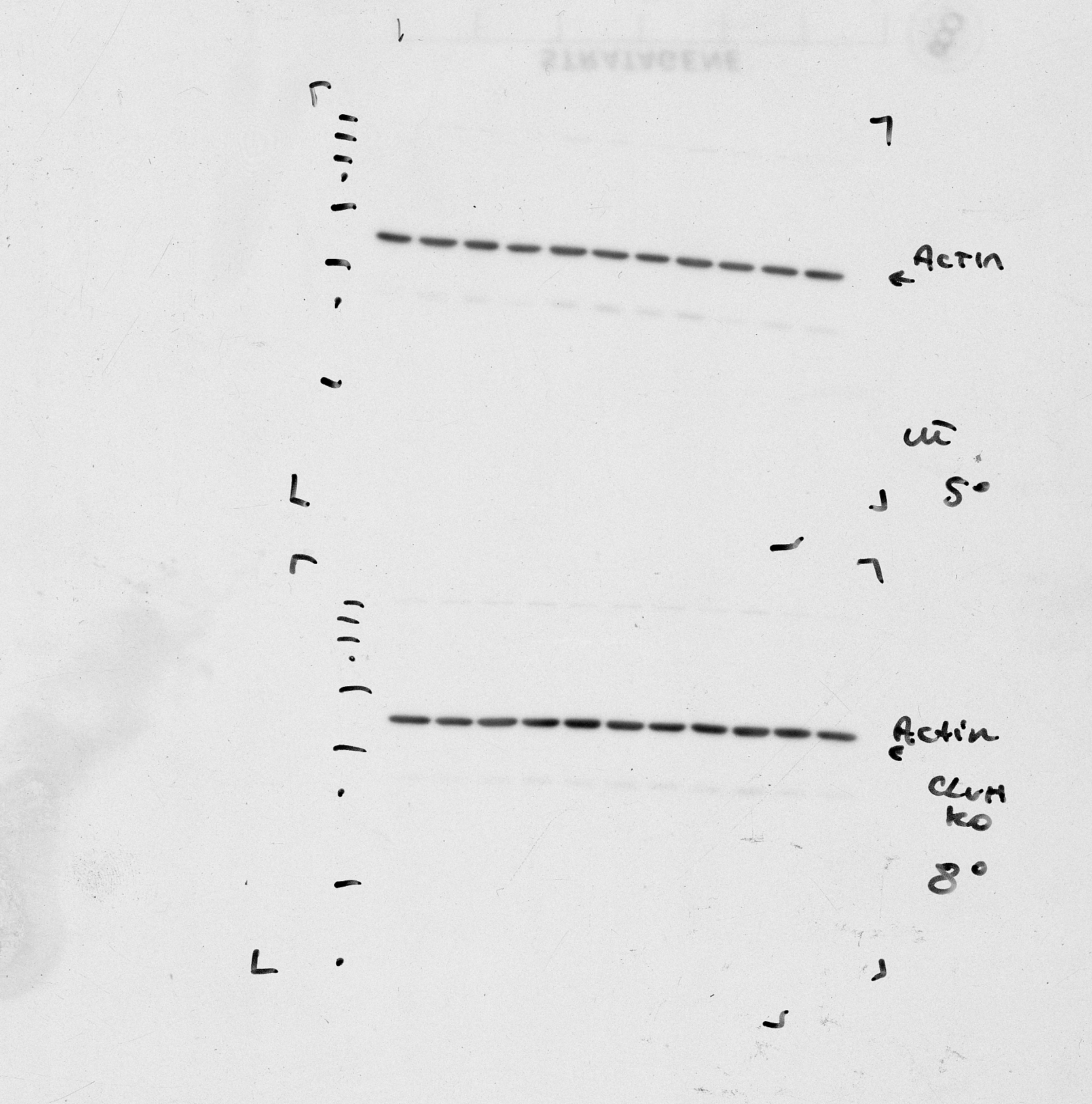

Supplement: Figure 7—source data 2. [file elife-74552-fig7-data2.zip › Figure 7ΓÇösource data 2/Uncropped blots for Figure 7H/kinastrin actin.tif]

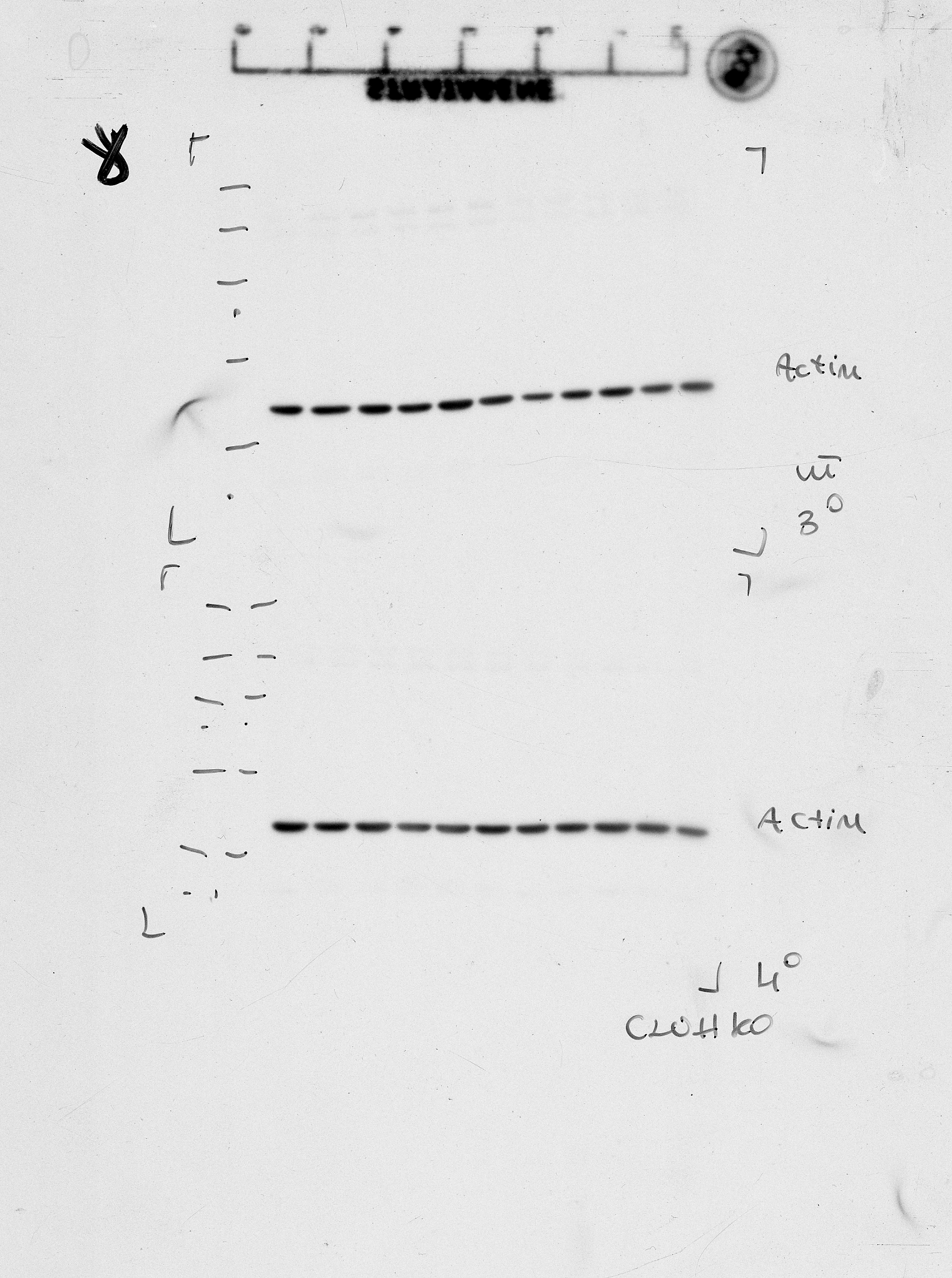

Supplement: Figure 7—source data 2. [file elife-74552-fig7-data2.zip › Figure 7ΓÇösource data 2/Uncropped blots for Figure 7H/astrin CDK1 actin.tif]

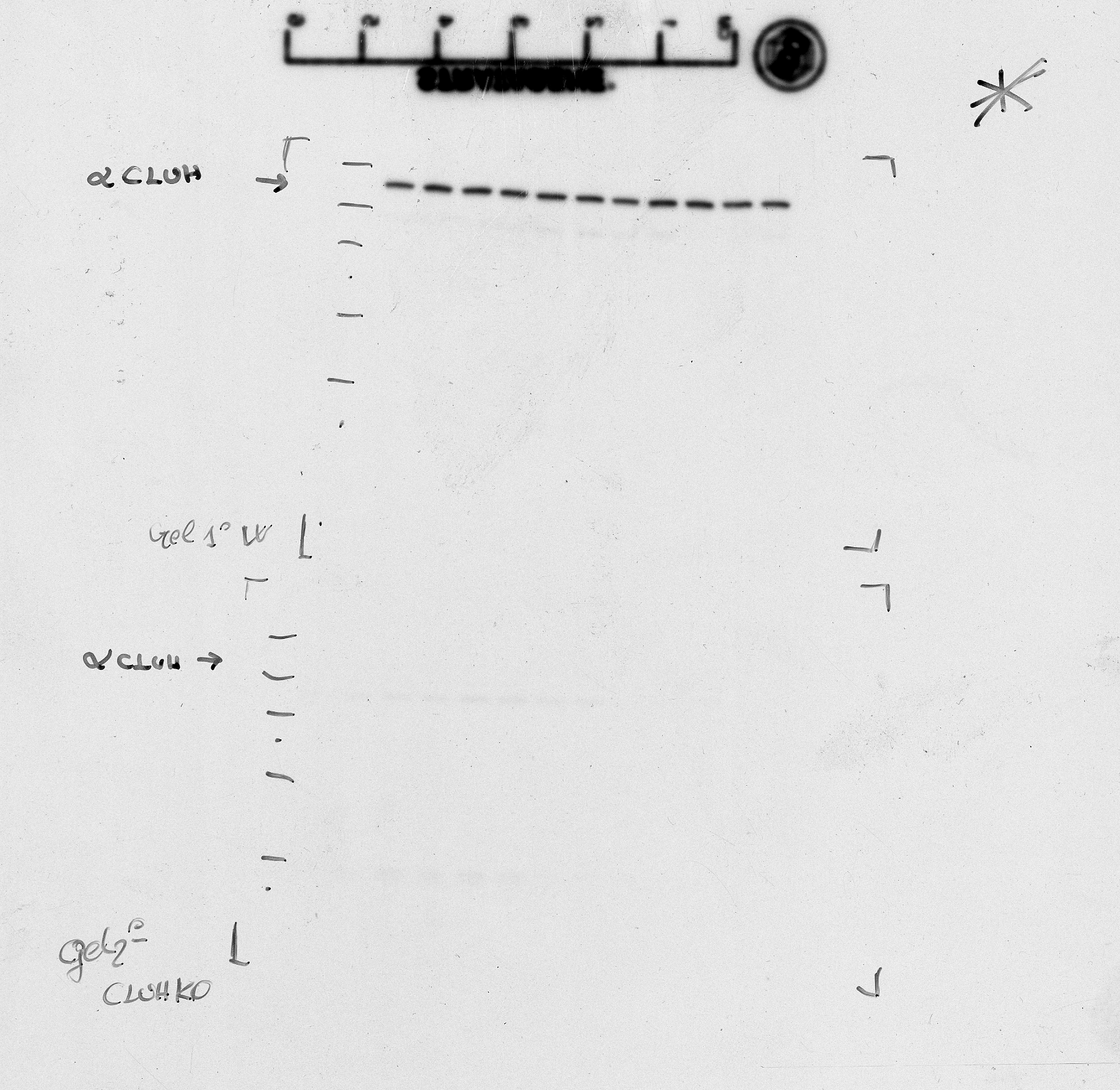

Supplement: Figure 7—source data 2. [file elife-74552-fig7-data2.zip › Figure 7ΓÇösource data 2/Uncropped blots for Figure 7H/CLUH.tif]

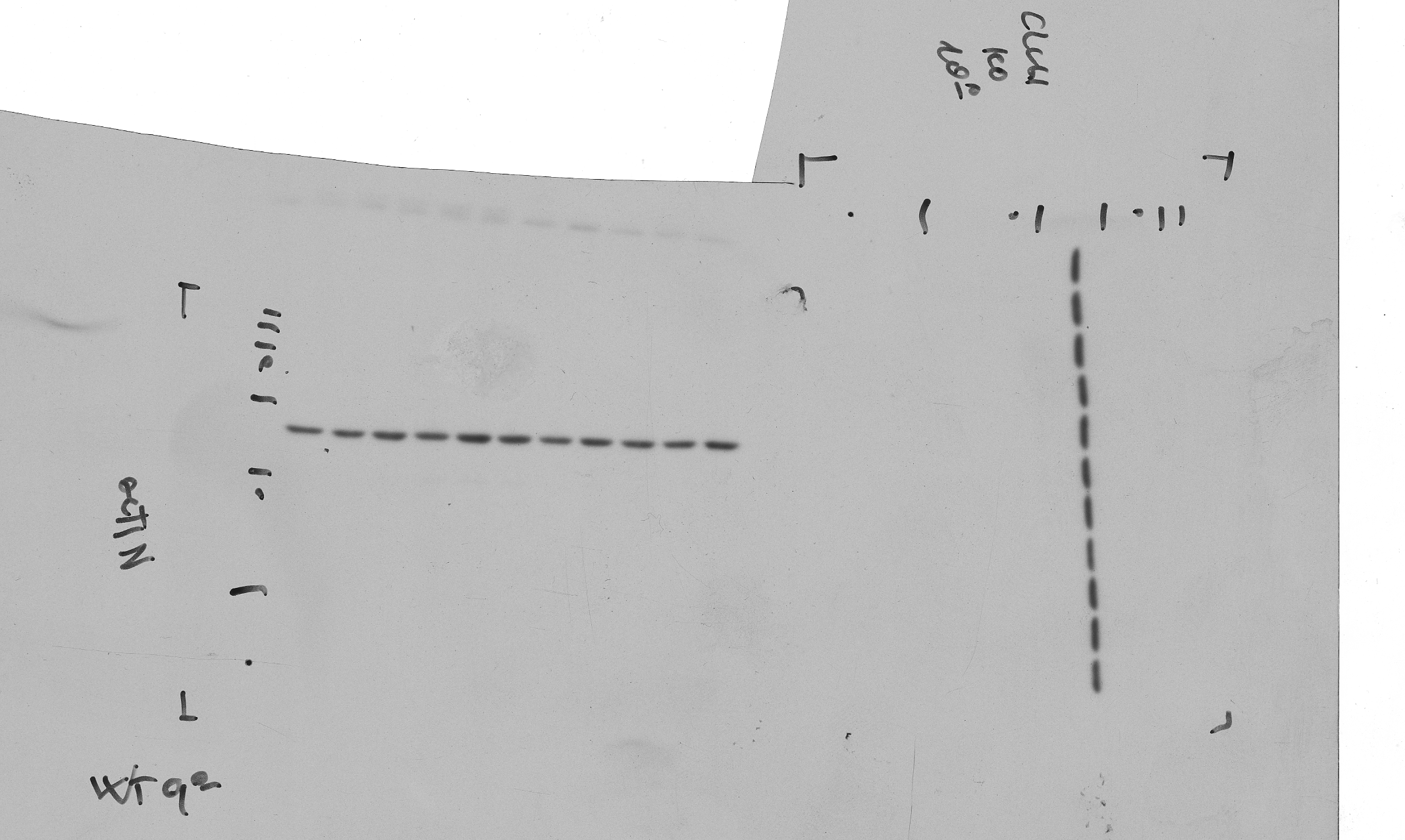

Supplement: Figure 7—source data 2. [file elife-74552-fig7-data2.zip › Figure 7ΓÇösource data 2/Uncropped blots for Figure 7H/pH3 actin.tif]

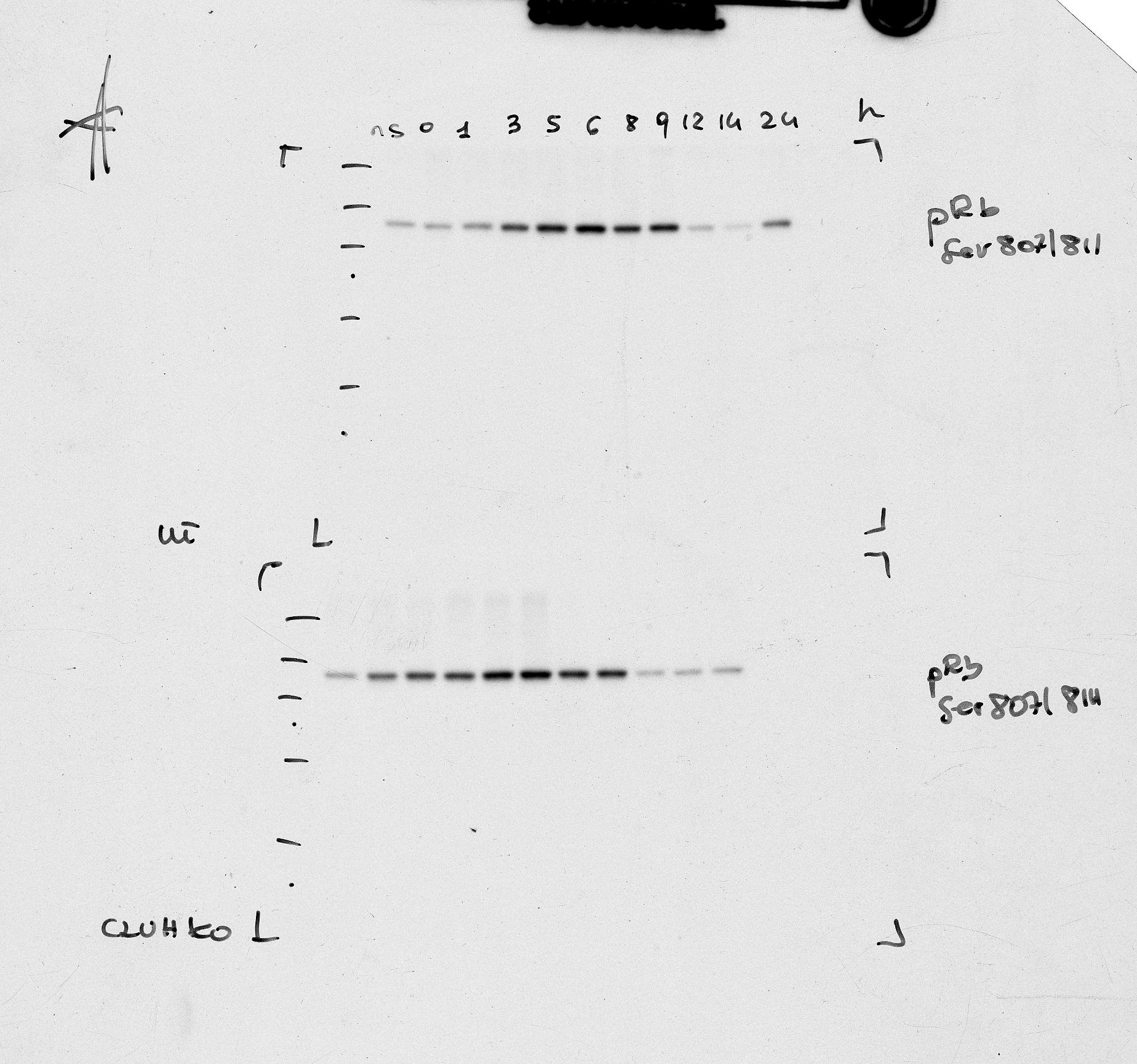

Supplement: Figure 7—source data 2. [file elife-74552-fig7-data2.zip › Figure 7ΓÇösource data 2/Uncropped blots for Figure 7H/pRB1-Ser807811.tif]

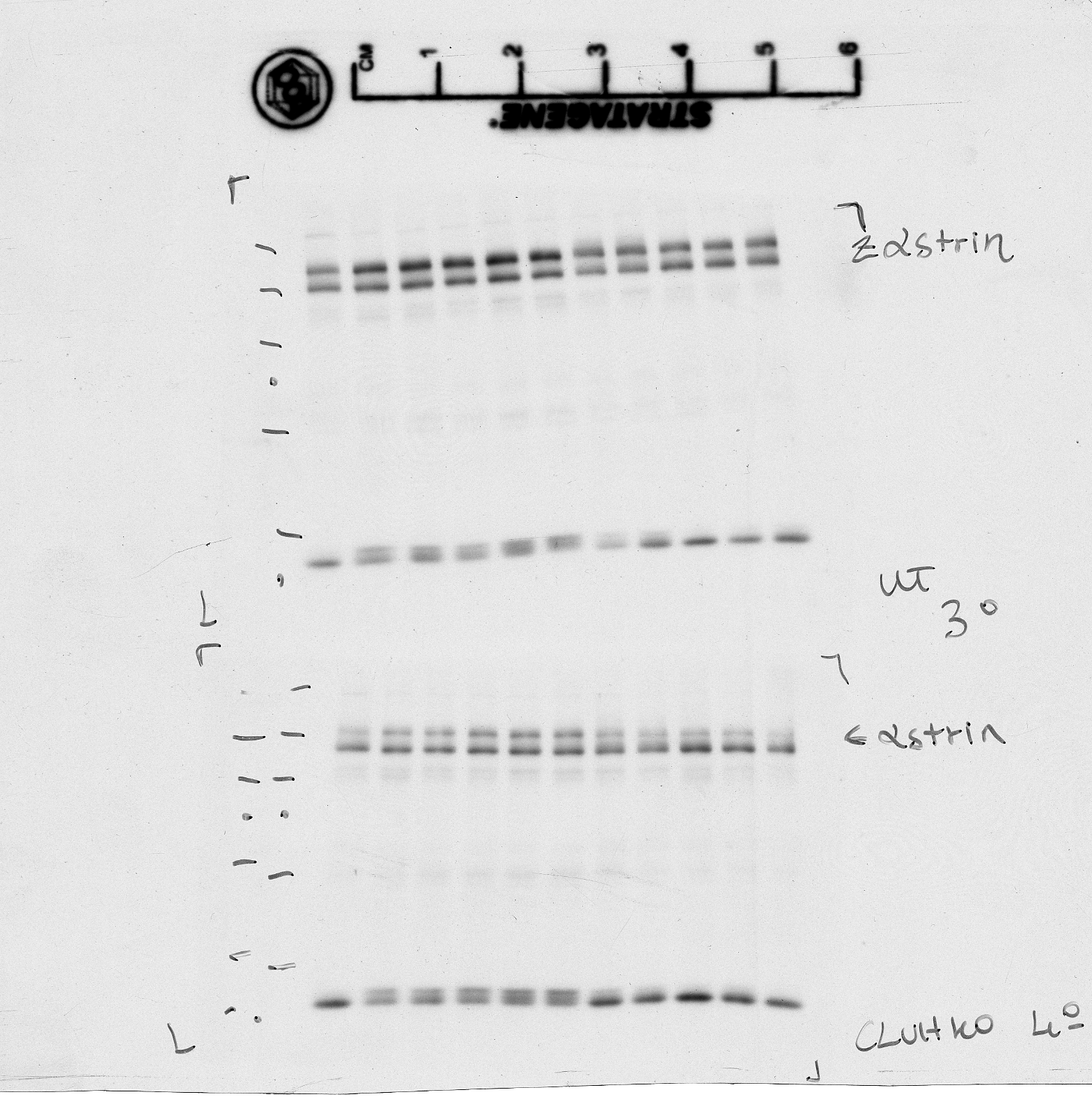

Supplement: Figure 7—source data 2. [file elife-74552-fig7-data2.zip › Figure 7ΓÇösource data 2/Uncropped blots for Figure 7H/astrin.tif]
