# Supplementary material for: CLUH controls astrin-1 expression to couple mitochondrial metabolism to cell cycle progression
Source: eLife. 2022 May 13;11:e74552. doi: 10.7554/eLife.74552 (PMC9135405; doi:10.7554/eLife.74552)
Supplement: Figure 4—source data 2. [file elife-74552-fig4-data2.zip › Figure 4-source data 2/SDHA.pdf]

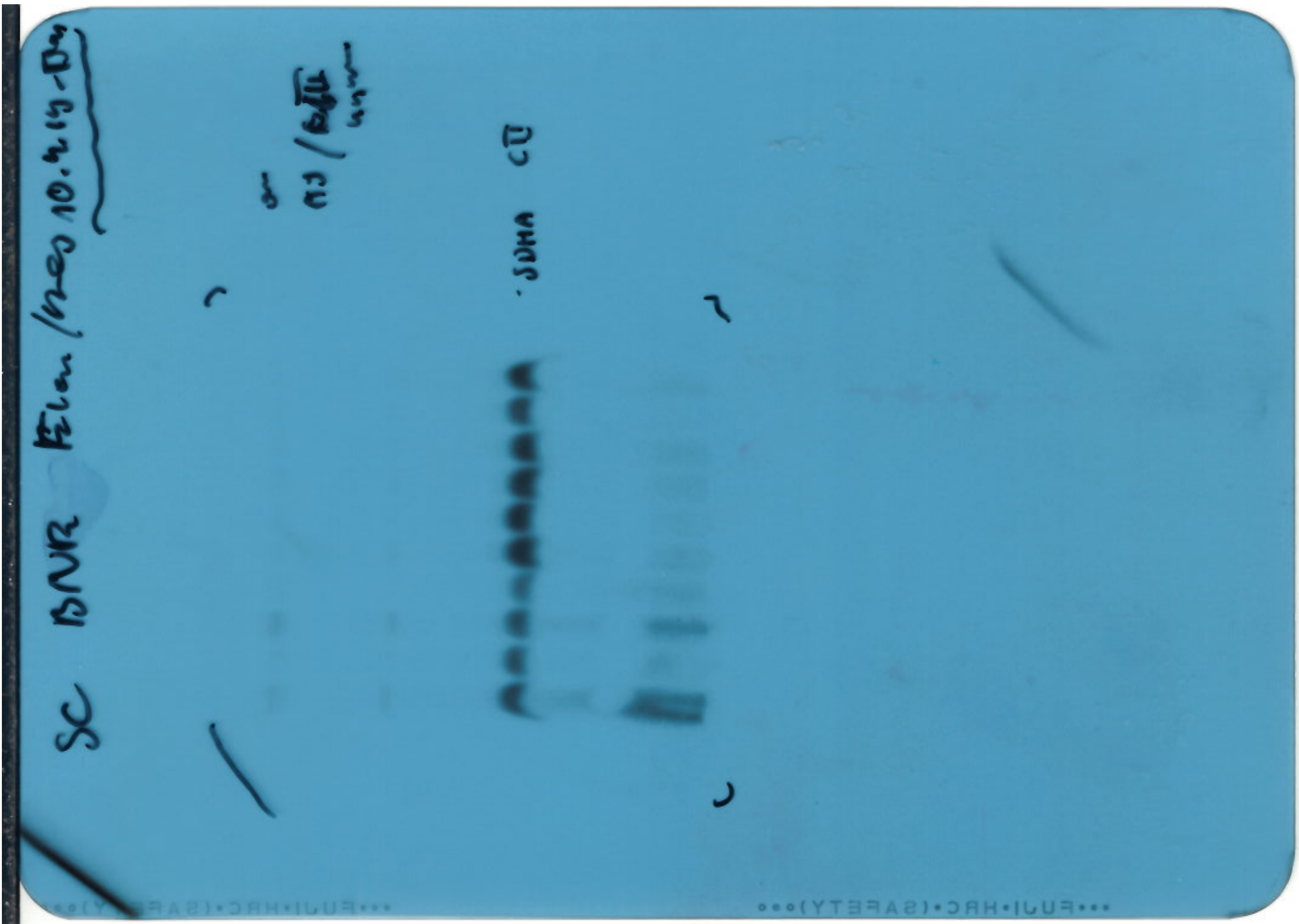
SC BNR
